# High sensitivity and low-cost flavin luciferase (FLUX^Vc^)-based reporter gene for mammalian cell expression

**DOI:** 10.1016/j.jbc.2023.104639

**Published:** 2023-03-24

**Authors:** Jittima Phonbuppha, Ruchanok Tinikul, Yoshihiro Ohmiya, Pimchai Chaiyen

**Affiliations:** 1 School of Biomolecular Science & Engineering, Vidyasirimedhi Institute of Science and Technology (VISTEC), Rayong, Thailand; 2 Department of Biochemistry and Center for Excellence in Protein and Enzyme Technology, Faculty of Science, Mahidol University, Bangkok, Thailand; 3 National Institute of Advanced Industrial Science and Technology (AIST), Ikeda, Osaka, Japan; 4 Osaka Institute of Technology (OIT), Osaka, Osaka, Japan

**Keywords:** bacterial luciferase, flavin, reporter gene, bioluminescence, cell signaling, NF-κB signaling pathway, polyphenol

## Abstract

Luciferase-based gene reporters generating bioluminescence signals are important tools for biomedical research. Amongst the luciferases, flavin-dependent enzymes use the most economical chemicals. However, their applications in mammalian cells are limited due to their low signals compared to other systems. Here, we constructed Flavin Luciferase from *Vibrio campbellii* (*Vc*) for Mammalian Cell Expression (FLUX^Vc^) by engineering luciferase from *V. campbellii* (the most thermostable bacterial luciferase reported to date) and optimizing its expression and reporter assays in mammalian cells which can improve the bioluminescence light output by >400-fold as compared to the nonengineered version. We found that the *FLUX*^*Vc*^ reporter gene can be overexpressed in various cell lines and showed outstanding signal-to-background in HepG2 cells, significantly higher than that of firefly luciferase (Fluc). The combined use of FLUX^Vc^/Fluc as target/control vectors gave the most stable signals, better than the standard set of Fluc(target)/Rluc(control). We also demonstrated that FLUX^Vc^ can be used for testing inhibitors of the NF-κB signaling pathway. Collectively, our results provide an optimized method for using the more economical flavin-dependent luciferase in mammalian cells.

High throughput screening technology is important for biomedical research because it can be employed to find drug candidates against infectious and noncommunicable diseases. Noncommunicable diseases are chronic diseases not transmissible between people and have become the world’s major killers, accounting for 71% of all deaths globally (1). Despite the rapid developments in screening technology which enable new drugs or active pharmaceutical ingredients to be discovered, such resources are not widely accessible around the world, and are especially lacking in developing countries due to the high costs. This has resulted in the majority of low- to middle-income countries (LMICs) suffering from high numbers of premature deaths, mainly in the South-East Asian, Eastern Mediterranean, and African regions (2). Over 80% of the overall deaths in LMIC regions are caused by cardiovascular, (lung, esophagus, stomach, and liver) cancers, chronic respiratory, congenital birth defects, and digestive diseases (2, 3). Therefore, development of effective and affordable high-throughput assays or screening (HTS) tools would be beneficial and facilitate the distribution of sustainable scientific advancements across the globe.

Reporter genes are among the most common tools used for drug and bioactive compound screening. The technique can be used for monitoring cellular events associated with signal transduction, gene expression as well as disease progression (4, 5). Reporter genes can generate various signals for detection including absorbance, fluorescence, and luminescence. Among these detection methods, luminescence is the most sensitive techniques, providing detection limit as low as 10^−18^ to 10^−21^ mol of analytes (5, 6). Unlike fluorescence detection which requires excitation light that can create high background, bioluminescence does not require light input, thus eliminating photobleaching and lowering background signals. Bioluminescence usually generates a broad linear range of detection as well as high sensitivity (5, 7). It is widely used for monitoring cell progression, cytotoxicity, gene expression, and cellular events relevant to a regulatory element, transcription factors, and activities of bioactive compounds (7, 8, 9, 10).

Bioluminescence is catalyzed by an enzyme class called luciferases, which is present in various organisms. Different luciferases use different substrates or luciferins with a common use of oxygen to generate light with specific wavelengths and signals. Some reported luciferin and luciferase systems include (1) coelenterazine substrates for *Renilla*, *Gaussia*, and *Oplophorus* luciferases, (2) D-luciferin substrate for firefly and Click beetle luciferases, (3) flavin-dependent system for bacterial luciferase, (4) cypridina luciferin-based system for *Cypridina* and *Porichthys* luciferases, (5) tetrapyrrole-based luciferins for luciferases from Dinoflagellates and Euphausiids, and (6) 3-hydroxyhispidin-based system for fungal luciferase (Fig. S1) (11). The first two groups of luciferins/luciferases are among the most commonly used systems as gene reporters because their light signals are high, giving high sensitivity in detection applications (Fig. S1, *A* and *B*). Currently, the D-luciferin and firefly luciferase (Fluc) is the most widely used system which gives good signals and robustness for its applications (Fig. S1*B*). However, this reporter system has limitations in screening plant-derived compounds especially polyphenols or isoflavonoids because these compounds interfere with Fluc activities (12, 13). Although the coelenterazine system is simpler than other systems because it only requires one substrate plus oxygen (Fig. S1*A*), the compound is unstable and can release photons even in the absence of the *Renilla* luciferase (Rluc), resulting in relatively high background (14, 15). The coelenterazine/*Rluc* system is also quite expensive; its price per assay is ∼1.4-times that of the D-luciferin/Fluc. Tetrapyrrole-based system is highly active under acidic conditions and the substrate is not commercially available (Fig. S1*E*), while the 3-hydroxyhispidin luciferase has just recently been discovered and still requires further investigation to fully understand its mechanisms (Fig. S1*F*). Among all existing luciferase systems, the flavin-dependent enzyme uses the simplest (thus most economical) substrates in (Fig. S1*C*) which the price per assay would be 1/100 of the firefly system. However, its applications as a reporter gene in mammalian cells are limited due to its low signal. The successful development of the flavin-dependent luciferase as a gene reporter assay in mammalian cells would contribute to technology enabling high-throughput screening (HTS) tools that are ∼100-time and ∼150-time less expensive than the firefly and *Renilla* systems, respectively.

Bacterial luciferase (Lux) catalyzes bioconversion of reduced flavin, long chain aldehyde, and oxygen to result in oxidized flavin, long chain carboxylic acids, and H_2_O with concomitant light production with maximum emission around 490 nm. The enzyme consists of α and β subunits which are individually encoded by the genes *luxA* and *luxB*, respectively in the *lux* operon (16). The *lux* operon also contains the *luxCDE* genes encoding multienzyme fatty acid reductase complex (LuxCDE) that converts fatty acid to aldehyde to supply the Lux reaction (17). Several species of luminous bacteria also contain the luxG gene which encodes for a flavin reductase that catalyzes the production of reduced flavin (I), a substrate for Lux (18). Reduced flavin is the first substrate to bind to Lux (II), followed by the oxygen reaction to generate the C4a-peroxyflavin intermediate (III), which attacks an aldehyde substrate and generates the following C4a-peroxyhemiacetal intermediate (IV). The cleavage of the O–O bond in C4a-peroxyhemiacetal yields fatty acid and the excited C4a-hydroxyflavin (V), which emits the blue–green light with λ_max_ around 490 nm (Fig. S2) (16, 19, 20, 21, 22). In the past, several studies have investigated the expression of Lux in mammalian cells but most of them only investigated Lux from the terrestrial microbe, *Photorhabus luminescens* (*Pl*_Lux) (23, 24, 25, 26, 27, 28, 29, 30, 31, 32, 33). The results of these studies showed that mammalian cells can overexpress *Pl*_Lux but their signals are rather low (23, 26, 32, 33, 34). To the best of our knowledge, none of the studies has compared the analytical power of *Pl*_Lux to other commonly used luciferases side-by-side nor demonstrated that the *Pl*_Lux can be used in substitution of firefly or *Renilla* as a gene reporter.

We proposed that Lux from *Vibrio campbellii* (*Vc*_Lux) is another attractive system to be used as a gene reporter. *Vc*_Lux is the most thermostable bacterial luciferase reported to date and when expressed in *Escherichia coli* can generate about 100-fold brighter light than the enzyme from *Vibrio harveyi* (*Vh*_Lux) (16, 19, 35, 36). As *Vh*_Lux generates light 5-fold brighter than that of *Pl*_Lux (37), it can be assumed that light generated by *Vc*_Lux is much brighter than that of *Pl*_Lux. Although *Vc*_Lux with two subunits fused *via* a linker can be overexpressed in mammalian cells, the system previously constructed gives very low bioluminescent signals (35, 38), making it still impractical for gene reporter applications in mammalian cell systems.

In this work, we improved the expression level of the fusion *Vc*_LuxAB (Lux) by optimizing its codon usage and modified the cocktail assay reagent and cell lysis reagents for the enzyme assay. The newly developed Flavin Luciferase from *V. campbellii* (*Vc*) for Mammalian Expression (FLUX^Vc^) showed remarkable performance, yielding signals >400 times brighter than the system without optimization. These optimizations, for the first time, significantly increased the bioluminescence signals of the bacterial luciferase to be close to the firefly enzyme (only about 20-fold lower). Furthermore, the FLUX^Vc^ system has the added advantage of generating much lower background signal than Fluc or Rluc. We explored the use of FLUX^Vc^ in various cell lines including HEK293T, NIH3T3, COS1, and HepG2 cells in comparison to Fluc. The results showed that FLUX^Vc^ can be expressed well in all four types of cell lines and its signal-to-background (S/N) ratio is even higher than Fluc in HepG2 cells. As transient transfection of gene reporters generally requires two different types of light signal generators, one as the target vector for addressing experimental effects and another as the control vector for evaluating the transfection efficiency, we thus explored the use of three luciferases (Fluc, Rluc, and FLUX^Vc^) in this combined luciferase-reporter gene assays. We found that the combined use of FLUX^Vc^ as the target vector and Fluc as the control vector gave the best result. This combined FLUX^Vc^/Fluc luciferase-reporter gene assay was investigated for its sensitivity in investigating the effects of tumor necrosis factor (TNF)-alpha and inhibitors on the NF-κB cell signaling pathway. The results showed that the FLUX^Vc^/Fluc reporter system gave similar EC_50_ values compared to the use of Fluc/Rluc as target/control vectors, validating the potential use of FLUX^Vc^ in gene reporter applications (Fig. 1). As luciferase reporter systems are widely used in the biomedical community (In year 2020 alone, more than 22,000 publications used luciferase reporters, (Fig. S3, pink bars), the 1/100 price reduction of FLUX^Vc^ would make the system attractive as an alternative gene reporter with significant cost reduction, while maintaining good capability in HTS applications (particularly for many plant-derived compounds which are inhibitors for Fluc but not FLUX^Vc^) in the future.

**Figure 1:**
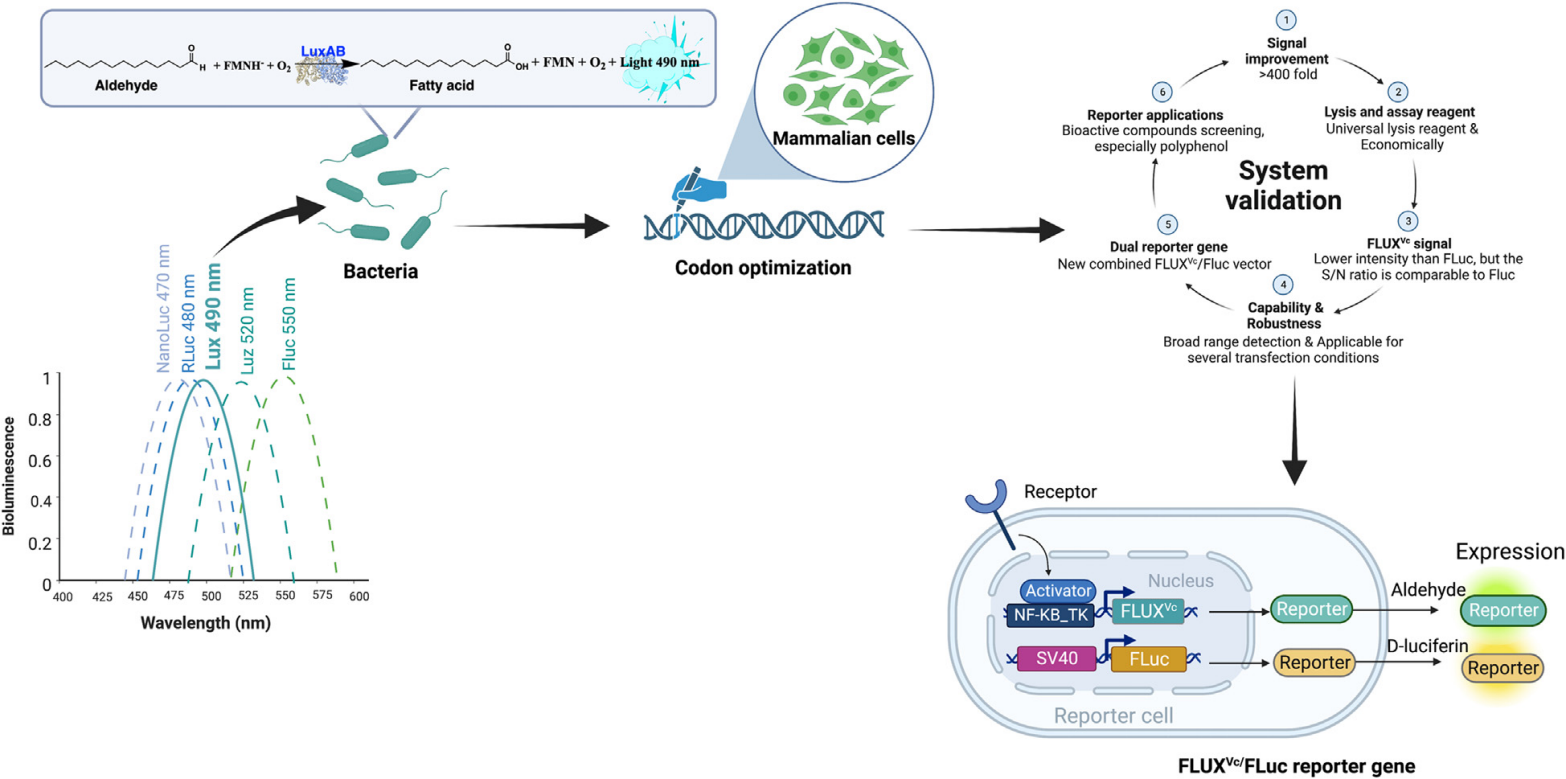
**Overall development, validation, and application concept of the FLUX^Vc^ reporter gene**.

## Results

### Optimization of a fusion bacterial luciferase (lux) gene expression in mammalian cells

**Figure 2:**
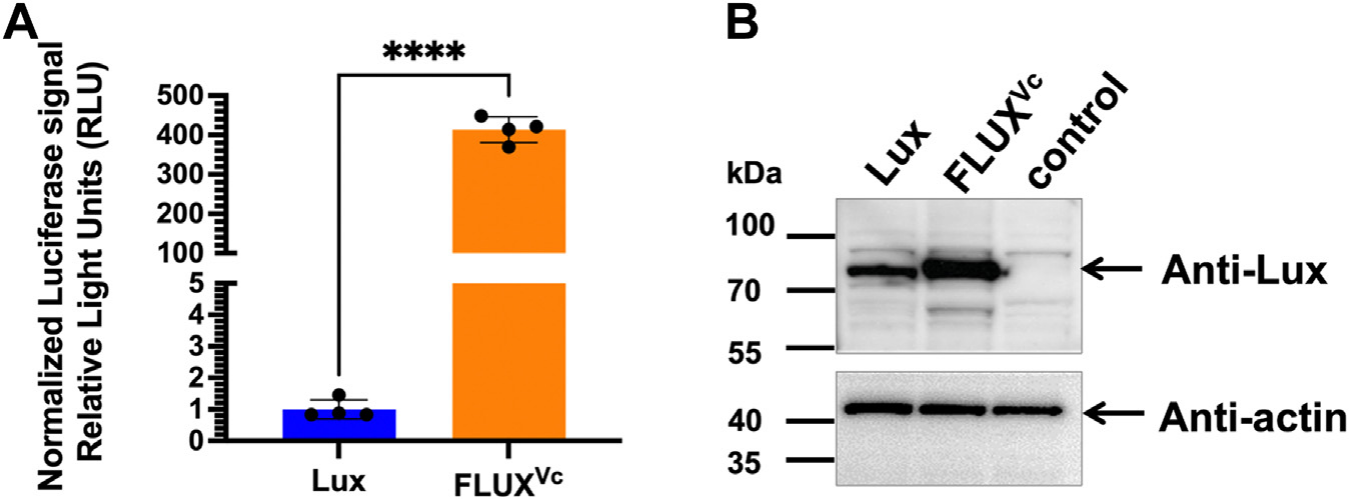
**Expression of the original fusion bacterial luciferase gene (*lux*) and codon optimized (*FLUX***^***Vc***^**) in HEK293T cells.** Vectors of pGL3 [*lux*/SV40] or pGL3[*FLUX*^*Vc*^/SV40] (0.07 pmol each) and 0.007 pmol of pRL-TK vector (internal control) were cotransfected into HEK293T cells. After 48 h of transfection, cells were collected, and Lux Lysis Reagent (LLR) was added, and the protein expression and luciferase activity were measured. *A*, the activity of Lux was monitored by adding a solution (100 μl) of reagent cocktail consisting of 5 μM FMN, 100 μM HPA, 10 μM decanal, and 100 μM NADH in 50 mM sodium phosphate buffer pH 7.0 into a solution of cell lysate that was freshly mixed with 50 mU of reductase C_1_. The luminescence signal was monitored for 10 s with a 2 s delay using an AB-2250 single tube luminometer. The *Rluc* activity was measured using the *Renilla* luciferase assay reagent according to the manufacturer’s instructions. The Lux activity was normalized with the Rluc activity and reported as normalized bioluminescence signals by comparing the fold change in activities. Data are presented as mean ± SD of four biological replicates. Student'*s t* test was used to evaluate significance. ∗∗∗∗ has *p* < 0.0001. *B*, expression level of each Lux was measured using Western blot analysis with antibody against the fusion luxAB for detection of Lux protein overexpressed in cells containing the Lux (pGL3[*lux*/SV40]) and FLUX^Vc^ (pGL3[*FLUX*^*Vc*^/SV40]). The control sample was lysate from cells without any transfection. Anti-actin was used for detection of actin as a housekeeping gene for normalizing the signals. Signal detection was carried out using secondary staining with HRP-conjugation and chemiluminescent reagents for HRP. The luminescence from HRP activity was imaged on a WSE-6100 LuminoGraph I Gel documentation system. FMN, flavin mononucleotide; HPA, *p*-hydroxyphenylacetic acid; HRP, horseradish peroxidase.

We first constructed and optimized a fusion Lux from *Vibrio cambellii* in which both α and β subunits are linked *via* an artificial linker obtained from modification of the intergenic sequence linking *luxA* and *luxB* by adding the nucleotide (**G**) upstream of the stop codon of *lux*A gene (TAA) and mutating the start codon of luxB from ATG to **CAG** in order to abolish the stop codon and avoid any internal initiation of translation of *lux*B. The sequence of the artificial linker is **G**TAATTAATATTTTCGAAAAG GAAAGAGAC**CAG**, which encodes for 11 amino acids (VINIFEKERDQ). This allows Lux to be overexpressed mono-cistronically in mammalian cells (35). The previously published sequence of the *lux* gene from *Vibrio campbeiill* (*Vc_lux*) (35) was analyzed for the Codon Adaptation Index (CAI) which is an index of the synonymous codon usage bias for a nucleotide sequence and quantifies codon usage similarity between a gene and reference sequences (39) using the GenScript Rara Codon Analysis Tool. The analysis showed that the original *lux* gene has a CAI value of only 0.67. We optimized the codon of the *lux* gene (designated as the *FLUX*^*Vc*^ gene) to obtain the CAI value of 0.88 (sequence shown in Fig. S4 and deposited in the National Center for Biotechnology Information database with GenBank number MZ393808) which should be suitable for expression in mammalian cells as a CAI value of higher than 0.8 is generally recommended for good gene expression. Expression of the *FLUX*^*Vc*^ gene using the pGL3 vector under a constitutive SV40 promoter was investigated in comparison with the original *lux* gene. Each system was also cotransformed with the pRL-TK vector to normalize transfection efficiency. The results showed that FLUX^Vc^ showed significant improvement in the bioluminescent signals, yielding a 400 ± 30-fold increase in bioluminescent signals compared to that of the native lux (Fig. 2*A*). We further investigated whether this signal increment was due to higher levels of protein expression using Western blot analysis. The Western blot results showed that the protein overproduced by the *FLUX*^*Vc*^ gene was also significantly higher than that of *lux* gene (Figs. 2*B* and S5). Our data indicate that with suitable codon usage, the FLUX^Vc^ system can generate light reasonably well in mammalian cells.

### Optimization of lysis and assay reagents for maximum light output

Lysis reagent is a buffer solution used for lysing cells and stabilizing proteins of interest. Ideally, lysis reagents should be mild, efficient in causing cell lysis and compatible with reagents used in downstream assays. The main additives in lysis reagents are mainly detergent, protease inhibitors and protein stabilizers in a suitable buffer (40). Commercially available reagents include Reporter Lysis Buffer (Promega), Luciferase Cell Lysis Reagent (Promega), and Passive Lysis Buffer (PLB, Promega). The first two groups of lysis reagent are suitable for Fluc, while PLB is a special reagent that can be used for both Fluc and Rluc (41). In this work, we developed a lysis reagent for the FLUX^Vc^ system based on common reagents available in the laboratory.

**Figure 3:**
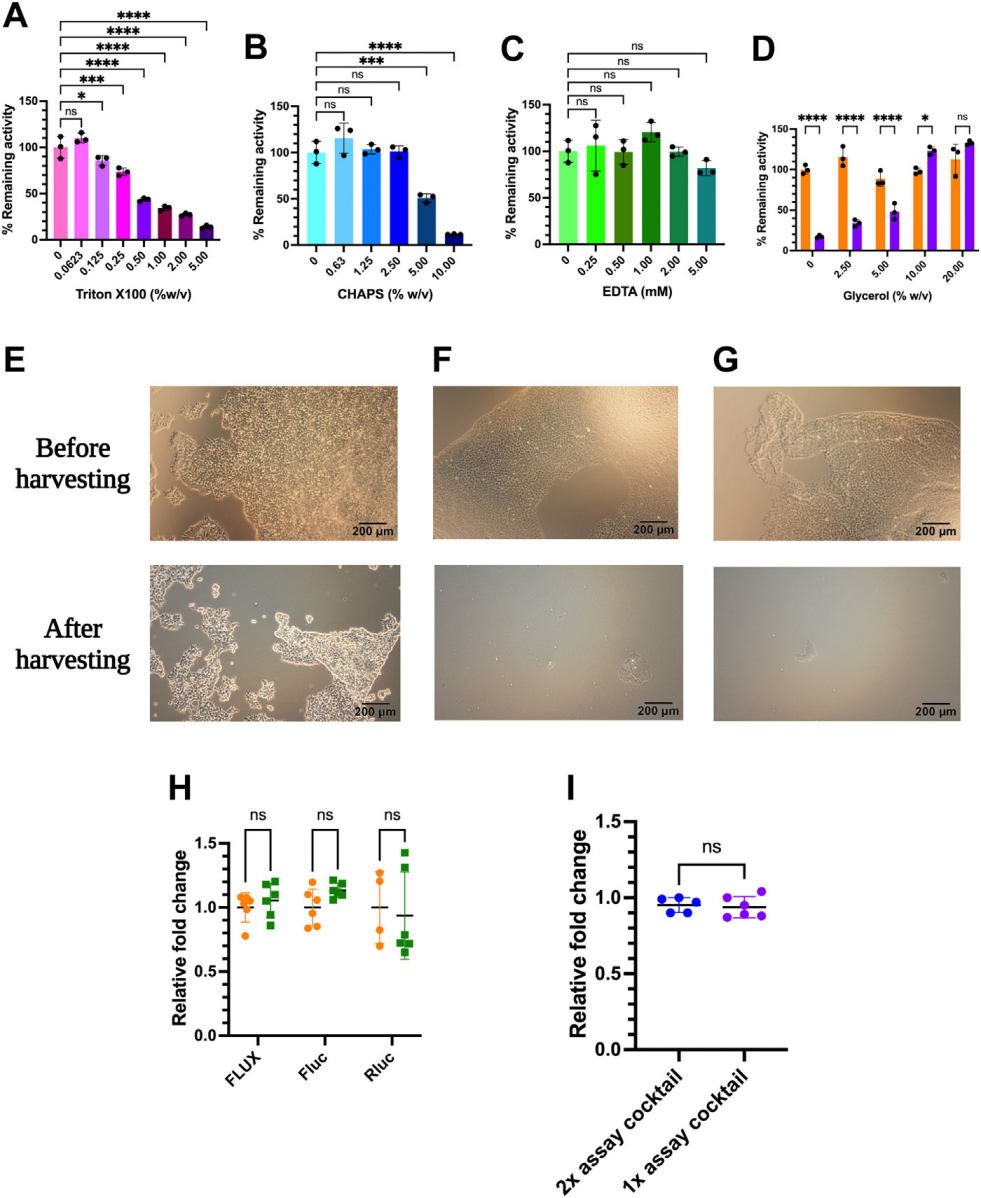
**Effects of various additive compounds on the purified Lux activity.***A*, Triton-X100, (*B*) CHAPS, (*C*) EDTA, and (*D*) glycerol before (*orange*) and after three freeze–thaw cycles (*purple*). Efficiency comparison of cell harvesting using different lysis reagents including (*E*) 50 mM sodium phosphate buffer pH 7.0, (*F*) Lux Lysis Reagent (LLR from the current report) and (*G*) Passive Lysis Buffer (PLB, Promega). *H*, comparison of effects of LLR and PLB on various luciferases including FLUX^Vc^, Fluc, and Rluc. *I*, FLUX^Vc^ activities from using 2x assay cocktail (*dark blue*) and 1x assay cocktail (*dark purple*). *A*–*D*, the effects of each additive on Lux activity was investigated by premixing the purified Lux solution with each additive at various concentrations and incubating for 15 min before measuring Lux activity compared to purified Lux. The activity of Lux was monitored by adding a solution (100 μl) of 1x assay cocktail consisting of 5 μM FMN, 100 μM HPA, 10 μM decanal, and 100 μM NADH in 50 mM sodium phosphate buffer pH 7.0 into a solution of incubated Lux that was freshly mixed with 50 mU of reductase C_1_. The luminescence signal was monitored for 10 s with a 2 s delay using an AB-2250 single tube luminometer. Each Lux activity was divided by the Lux activity measured in the absence of any additive compounds to obtain a remaining activity. The remaining activity was multiplied by 100 to obtain % remaining activity. Data are presented as mean ± SD of four biological replicates. ANOVA test was used to evaluate significance; ∗*p* < 0.05; ∗∗*p* < 0.01; ∗∗∗*p* < 0.001; ∗∗∗∗*p* < 0.0001. *E*–*I*, effects of various lysis reagents on cell detachment were investigated by plating 1 × 10^5^ HEK293T cells on a 24-well plate for 24 h before adding 100 μl of lysis reagent and then monitoring HEK293T cell morphology using an IX73 inverted light microscope. Cells were incubated at room temperature with rocking for 15 min before collecting the detached cells and the remaining HEK293T cells on the culture plate were examined using the IX73 inverted light microscope. *H* and *I*, cell lysate from transient transfection of target luciferase with internal control pRL-TK vector was used to investigate the effects of LLR (*orange*) and PLB (*green*) and Lux assay cocktail, respectively. Data are presented as mean ± SD of five biological replicates. Student's *t* test was used to evaluate significance; NS, not significant. FMN, flavin mononucleotide; HPA, *p*-hydroxyphenylacetic acid.

We aimed to find suitable reagents including lysis detergent, protease inhibitor, and protein stabilizing agents for the FLUX^Vc^ assays. First, Triton X-100 and 3-((3-cholamidopropyl) dimethylammonio)-1-propanesulfonate (CHAPS), which are nonionic and zwitterionic amphipathic compounds, respectively were used to lyse cells by solubilizing lipids and proteins in the membrane and creating pores within the membrane for full cell lysis. Their effects on FLUX^Vc^ activity were investigated by measuring the bioluminescent signals of the purified recombinant Lux in various detergent concentrations. The results showed that Lux activity was susceptible to TritonX-100 because even the lowest TritonX-100 concentration (0.125% w/v) resulted in a 10% decrease in activity of Lux, while 0.5% (w/v) TritonX-100 decreased the activity by 50% (Fig. 3*A*). The Lux activity is more stable in the presence of CHAPS, demonstrating stability in CHAPS concentrations ranging from 0.63 up to 2.5% (w/v), while 5% (w/v) CHAPS resulted in a drop-in activity of around 50% (Fig. 3*B*). These results suggest that 1 to 2%(w/v) of CHAPS is suitable for lysing the mammalian cells transfected with the *FLUX*^*Vc*^ reporter gene. For protease inhibitors, EDTA which is a metal chelator capable of chelating metal cofactors of several metalloproteases generally produced in mammalian cells was chosen as a protease inhibitor additive. We investigated the effects of EDTA on FLUX^Vc^ activity by measuring bioluminescence signals of the purified recombinant Lux in various concentrations of EDTA. The results showed that EDTA does not affect FLUX^Vc^ activity; the system retained nearly 100% of the bioluminescence in 0.25 to 5 mM EDTA (Fig. 3*C*).

Freezing samples is a practical method for long-term storage of crude lysates or proteins because activity assays generally cannot be performed on the same day. However, freezing and thawing can cause protein misfolding or inactivation. As glycerol is a common reagent which can prevent ice crystallization or ice-liquid interface formation (42, 43), we thus explored the effects of glycerol on Lux activity during freeze–thaw processes by measuring the bioluminescence signals of Lux before and after three freeze–thaw cycles. The results showed that glycerol (2.5–20% (w/v)) had no effect on Lux signals because it could retain nearly 100% of the Lux activity over the entire range of glycerol concentrations investigated (Fig. 3*D*, Orange). However, only 10 to 20% (w/v) of glycerol allowed retention of ∼100% of Lux activity after three freeze–thaw cycles (Fig. 3*D*, Purple). These results suggest that addition of at least 10% (w/v) glycerol is recommended for stabilizing the protein during the freezing-thawing process.

A final formula of lysis reagents for the *FLUX*^*Vc*^ reporter gene or Lux Lysis Regent (LLR) developed in this work consists of 1% (w/v) CHAPS, 1 mM EDTA, 10% (w/v) glycerol in 50 mM sodium phosphate buffer pH 7.0 which is typically used in the assay reaction. Thus, we further investigated the efficiency of LLR compared to a buffer without any additive reagent by monitoring cell morphology on the culture plate before and after harvesting the cells. Commercial lysis buffers for harvesting adherent cells such as PLB (Promega) were also compared to evaluate the efficiency of LLR. Results showed that most of the adherent cells could be harvested by LLR (Fig. 3*F*, after harvesting), while using a buffer without any additives resulted in most of the cells remaining adhered to the culture plate (Fig. 3*E*, after harvesting). The LLR harvesting efficiency is comparable to that of PLB (Fig. 3*G*, after harvesting). We further investigated effects of both lysis reagents on various luciferases. The results showed that both LLR and PLB did not interfere with FLUX^Vc^, Fluc, or Rluc activities, as all of them showed similar relative light signals when using any type of lysis buffer (Fig. 3*H*). These results clearly suggested that LLR developed in this work is a suitable lysis buffer to remove and lyse adherent cells without causing any interference to the luciferase activity.

Previously, FLUX^Vc^ activities could be assayed using the reagents consisting of 10 μM flavin mononucleotide (FMN), 200 μM *p*-hydroxyphenylacetic acid (HPA), 200 μM NADH, and 20 μM decanal in 50 mM sodium phosphate buffer pH 7.0 (designated as a 2x Lux assay cocktail) (35). In this work, we further optimized the cocktail assay reagents to minimize the cost, while maintaining high-bioluminescent signals. We found that all reagents could be used in 50% of the amount originally used in the 2x Lux assay cocktail. Thus, the 1x Lux assay cocktail consisting of 5 μM FMN, 100 μM HPA, 100 μM NADH, and 10 μM decanal in 50 mM sodium phosphate buffer pH 7.0 could be used to provide bioluminescent signals comparable to the previously used 2x Lux assay cocktail (Fig. 3*I*) which would significantly reduce costs. Altogether, our results indicate that the optimization of lysis and assay reagents could maximize bioluminescent output and minimize the FLUX^V*c*^-reporter gene assay cost.

### Comparison of light generated from FLUX^Vc^ and Fluc

To evaluate whether the FLUX^Vc^ system can be used as a gene reporter in transient transfection for probing molecular events in mammalian cells, we carried out experiments to measure light S/N ratios to compare the sensitivity of FLUX^Vc^ to the Fluc reporter gene, which is broadly used in a wide variety of applications. Bioluminescence readout signals generally contain target signals and nonspecific signals from background, stray light, and detector offset, etc. (44). Because signals are normally measured from bioluminescence generated under the control of a constitutive or inducible promoter, the background from nonspecific signals thus can be measured from bioluminescence generated in the absence of any activating element (45). Therefore, a S/N ratio, not an absolute signal value, is generally used as a parameter to represent measurement signals. Generally, signal can be measured from bioluminescence generated under the control of a constitutive or inducible promoter, while background can be measured from bioluminescence generated in the absence of any activating element (45). The S/N value can evaluate the strength of the promoter or measure the potency of compounds/metabolites under investigation such as inhibitors or inducers of the promoter (46). For comparison and benchmarking, we chose two vector systems commonly used for expression of the Fluc reporter gene, pGL3[*luc*+] and pGL4[*luc*2] vectors (Promega), to construct the expression vector of FLUX^Vc^ to compare with the bioluminescence signals of Fluc. The pGL4[*luc*2] vector is the newest series of Fluc reporter genes with a significantly improved signal to background ratio compared to the previous version pGL3[*luc+*] vector (45). However, the pGL3[*luc+*] vector is still widely used in biological research, with 5040 published articles in 2020 (Fig. S3, green bars). Therefore, the *FLUX*^*Vc*^ gene was inserted into the pGL3 and pGL4 vectors downstream of the constitutive SV40 promoter as well as in the same position in a vector without SV40 promoter to construct vector sets with and without SV40 (Fig. S6). All systems were independently cotransfected with the vector containing Rluc as an internal control vector in various cell types including HKE293T, NIH3T3, COS1, and HepG2 cells (see full name in Experimental procedures).

**Figure 4:**
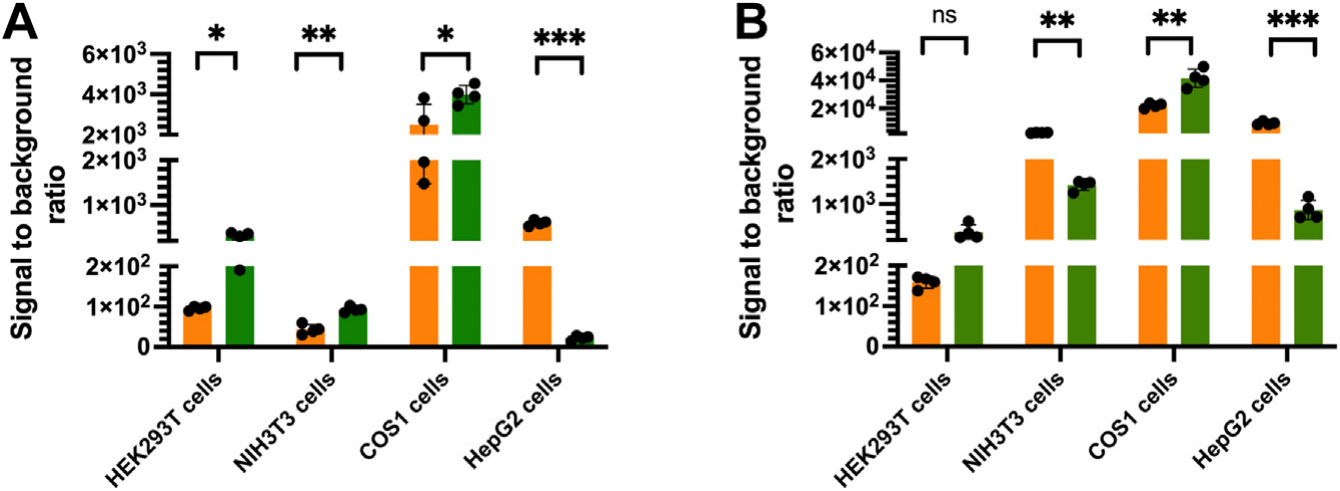
**Comparison of signal to background ratios of FLUX**^**Vc**^**(*orange*) and Fluc (*green*)-gene reporter systems.***A*, systems constructed in the pGL3 backbone vector and (*B*) Systems constructed in the pGL4 backbone vector. Various cell types including HEK293T, NIH3T3, COS1, and HepG2 cells were used for testing bioluminescence signals. HEK293T, NIH3T3, COS1, and HepG2 cells were cotransfected with each reporter gene (0.07 pmol) and the internal control pRL-TK vector (0.007 pmol). Cells were collected at 48 h after transfection by washing the culture with Passive Lysis Buffer (PLB) or Lux Lysis Reagent (LLR). Then, FLUX^Vc^ activity was measured by adding a 100 μl of assay cocktail consisting of 5 μM FMN, 100 μM HPA, 10 μM decanal, and 100 μM NADH in 50 mM sodium phosphate buffer pH 7.0 into a solution of cell lysate that freshly mixed with 50 mU of reductase C_1_. The luminescence signal was monitored for 10 s with a 2 s delay using an AB-2250 single tube luminometer. The Fluc and Rluc activities were measured using firefly luciferase and *Renilla* Luciferase Assay reagents, respectively according to the manufacturer’s instructions. The luciferase activity under the constitutive SV40 promoter was divided by their Rluc activity to obtain the normalized luciferase activity. The normalized signal of SV40 promoter vector was divided by the normalized signal from the corresponding promoterless vector to obtain a value of signal to background ratio. Data are presented as mean ± SD of four biological replicates. Student's *t* test was used to evaluate significance; ∗*p* < 0.05; ∗∗*p* < 0.01; ∗∗∗*p* < 0.001; NS, not significant. FMN, flavin mononucleotide; Fluc, firefly luciferase; HPA, *p*-hydroxyphenylacetic acid; Rluc, *Renilla* luciferase.

The results showed that the FLUX^Vc^ system generated less light intensity both in target and background signals than Fluc in both pGL3[*luc+*] and pGL4 [*luc2*] vectors (Fig. S7). This is due to the nature of the low quantum yield of Lux compared to Fluc (47, 48). However, analysis of S/N showed that S/N values of FLUX^Vc^ and Fluc expressed in the pGL3 expression vector are comparable in NIH3T3 and COS1 cells (Fig. 4*A*). S/N values of FLUX^Vc^ are less than the Fluc in HEK293T cells while they are considerably higher than Fluc in HepG2 cells (Fig. 4*A*). These S/N ratio behaviors are similar to the experiments comparing the FLUX^Vc^ and Fluc expression using the pGL4 vector (Fig. 4*B*). The higher S/N ratio of FLUX^Vc^ in HepG2 cells than Fluc is caused by the higher background signals of Fluc than FLUX^Vc^, whereas the two systems generated comparable target signals (Fig. S7). The higher background signals in the Fluc system (in both *luc*+ and *luc*2 genes) might be the result of anomalous expression of the reporter gene caused by cryptic regular-binging site or/and enhancer element (45). With the origin of FLUX^Vc^ being from bacteria, such anomaly is less evident in mammalian systems.

These data in Figure 4 clearly suggest that FLUX^Vc^ functioned well as gene reporters in HepG2 and COS1 cells because the S/N values of the FLUX^Vc^ systems were quite high in both cells with both vector types. For the NIH3T3 cells, the FLUX^Vc^ system also showed higher S/N values than the Fluc system with the pGL4 vector, also implying that the FLUX^Vc^ system should be able to serve as a gene reporter in NIH3T3 as well. Among all cell types, the FLUX^Vc^ system gave lower S/N ratio than the Fluc system in HEK293T cells (about 2-folds). Based on these data in Figure 4 alone, the ability of FLUX^Vc^ to serve as a gene reporter system in HEK293T cells was questionable. We thus investigated the ability of FLUX^Vc^ to serve as a gene reporter in HEK293T cells in the following sections. We chose to validate the function of FLUX^Vc^ in HEK293T cells to prove the functions of FLUX^Vc^ in the least favorable expression systems. If FLUX^Vc^ can be used as a gene reporter in HEK293T, the results inevitably endorse the use of FLUX^Vc^ in more favorable HepG2, COS1, and NIH3T3 cells.

### Exploring capability and robustness of the FLUX^Vc^-gene reporter system

To evaluate the use of FLUX^Vc^ system as a gene reporter, we explored the range of linear detection for FLUX^Vc^ to evaluate the sensitivity and working range of the system and also investigated parameters that can affect the transient transfection process including cell growth period and amount of vector used for transfection.

**Figure 5:**
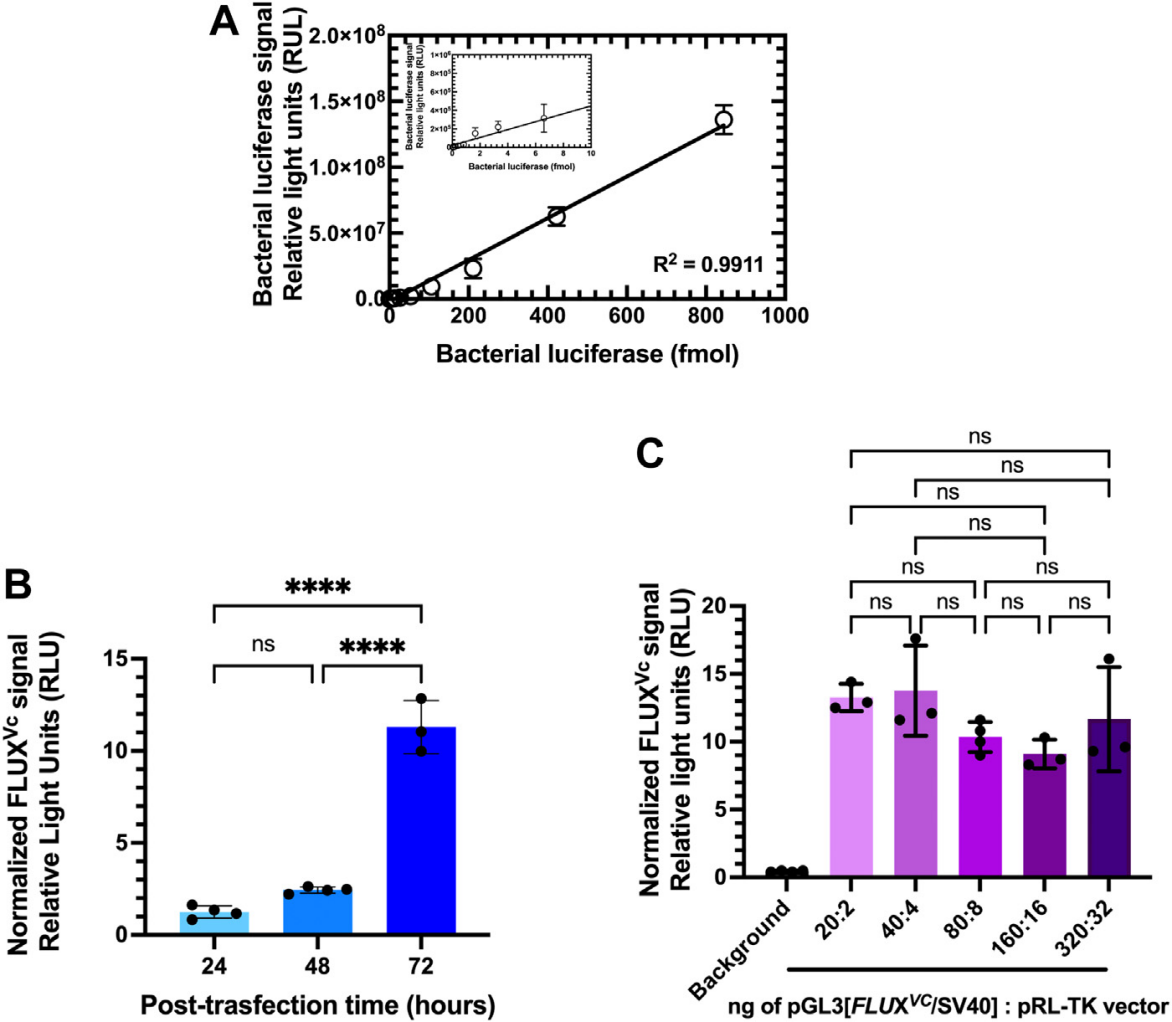
**FLUX**^**Vc**^**signals and their influencing factors.***A*, a correlation of responsive signals and the amount of purified recombinant Lux. Two-fold dilutions of Lux were made from 850 fmol to 25 amol for determining a correlation between Lux signal at various amount of the enzyme. The correlation of responsive signals with amount of Lux was plotted and shown in the insert figure. The coefficient of determination (R^2^) was analyzed by GraphPad Prism Version 9 software (https://www.graphpad.com) shown in the figure. *B*, effects of a post-transfection period on FLUX^Vc^ signals. HEK293T cells were transfected with the pGL3 [*FLUX*^*Vc*^/SV40] vector (0.07 pmol) and 0.007 pmol of the internal control pRL-TK vector. Cells were collected at 24, 48, and 72 h post-transfection using Lux Lysis Reagent (LLR) and the resulting luciferase activities were independently measured. *C*, normalized FLUX signals at various amounts of *FLUX* vector. A pGL3[*FLUX*^*Vc*^/SV40] vector was cotransfected with pRL-TK vector as internal control vector at target:control vector amount of 20:2, 40:4, 80:8, 160:16, and 320:32 ng into HEK293T cells. The activity of Lux was monitored by adding a 100 μl of assay cocktail consisting of 5 μM FMN, 100 μM HPA, 10 μM decanal, and 100 μM NADH in 50 mM sodium phosphate buffer pH 7.0 into a solution of cell lysate that was freshly mixed with 50 mU of reductase C_1_. The luminescence signal was monitored for 10 s with a 2 s delay using an AB-2250 single tube luminometer. The Rluc activity was measured using *Renilla* Luciferase Assay Reagent according to the manufacturer’s instructions. The FLUX^Vc^ activity was divided by their Rluc luciferase activity to obtain normalized luciferase signals. Data are presented as mean ± SD of four biological replicates. ANOVA test was used to evaluate significance; ∗∗∗∗*p* < 0.0001; NS, not significant. FMN, flavin mononucleotide; HPA, *p*-hydroxyphenylacetic acid.

First, the sensitivity and linear range of detection by FLUX^Vc^ were investigated by measuring the bioluminescence signals generated by various amount of the purified Lux. The results showed a broad linearity range with at least eight orders of magnitude; this is equivalent to a broad dynamic range of 0.25 fmol to 850 fmol (Fig. 5*A*). These data indicate that bioluminescent signals directly depend on the amount of Lux over a wide range, also enabling limits of detection down to a few molecules at attomole levels (10^−18^ mol). The wide range of FLUX^Vc^ detection limit (eight orders of magnitude) is similar to the detection range of Fluc and is wider than that of Rluc (about seven orders of magnitude) (41). However, Fluc and Rluc give higher sensitivity than FLUX^Vc^ because their limit of detection (LoD) is lower. The LoDs of Fluc, Rluc, and Lux are 0.01, 0.3, and 25 amol of enzymes, respectively which correlate with their quantum yields (0.48, 0.05, and 0.16 for Fluc, Rluc, and Lux, respectively) and their catalytic turnovers (1.6 s^−1^, 1.9 s^−1^, and 0.005 s^−1^, for Fluc, Rluc, and Lux, respectively) (16, 41, 47, 49, 50, 51, 52, 53). However, it should be noted that this LoD level was based on the purified enzymes which is not directly relevant to the FLUX^Vc^ expression in mammalian cells. We later showed that transfection of 10^5^ cells, a level typically used in biological research with 20 ng of the FLUX^Vc^ vector can yield good signals for practical experiments (Fig. S9 and see more results below).

Second, we investigated signals generated by FLUX^Vc^ after transfection. Transfected cells are typically harvested for analyzing levels of gene expression 24 h and up to 72 h post-transfection. A suitable time for cell harvesting is different, depending on cell type, research goals, and specific expression characteristics (54). Therefore, normalized FLUX^Vc^ activities in HEK293T cells transfected with the pGL3[FLUX^Vc^/SV40] vector and the pRL-TK vector as an internal control at various time points after transfection were measured. Results showed that the luciferase activities in cell lysate increased over time (Fig. 5*B*). The FLUX^Vc^ signal at 24 h post-transfection was significantly higher than the nontransfected cells by at least three orders of magnitude and was much higher than the background signal (Fig. S8, orange). The bioluminescent signals at 48 and 72 h post-transfection were 2-fold and 9-fold higher than that at 24 h post-transfection, respectively (Fig. 5*B*). The results suggested that although the signal from 24 h post-transfection was sufficient for typical measurement, a longer period such as 48 or 72 h could increase the signal significantly. The higher signals upon longer post-transfection times indicate that Lux is stable inside the cells and could accumulate signals upon prolonged cell growth. The stability of the intracellular FLUX^Vc^ protein makes the system suitable for HTS applications.

Third, we explored the suitable amount of vector required for transfection. Typically, a 96-well culture plate is commonly used for HTS applications and the amount of vector of 100 ng is normally recommended for transfection per well. We investigated the effects of the vector amount used for transfection on the FLUX^Vc^ signals using FLUX^Vc^/Rluc combination as a model for investigation. The amount of FLUX^Vc^ and Rluc reporter genes was varied from 20:2 ng to 320:32 ng, covering a range recommended for transfection and maintaining a final ratio of 10:1. The transfected cells were harvested at 24 h post-transfection and the bioluminescent signals were measured in cell lysate. The results showed that even at the lowest amount of the pGL3[FLUX^Vc^/SV40] vector (20 ng), a high signal could be obtained (Fig. S9, orange,). Both FLUX^Vc^ and Rluc signals could be increased directly according to the amount of transfected vector (Fig. S9, orange, and purple, respectively). Results in Figure 5*C* showed that the normalized FLUX^Vc^ signals which were obtained from dividing the original FLUX^Vc^ with Rluc signals were similar at all ratios investigated. The data suggest that FLUX^Vc^ could be used as a target reporter gene with requirement of only 20 ng per transfection.

### Combined use of FLUX^Vc^, Fluc, and Rluc as target/control vectors for luciferase-reporter gene assays

**Figure 6:**
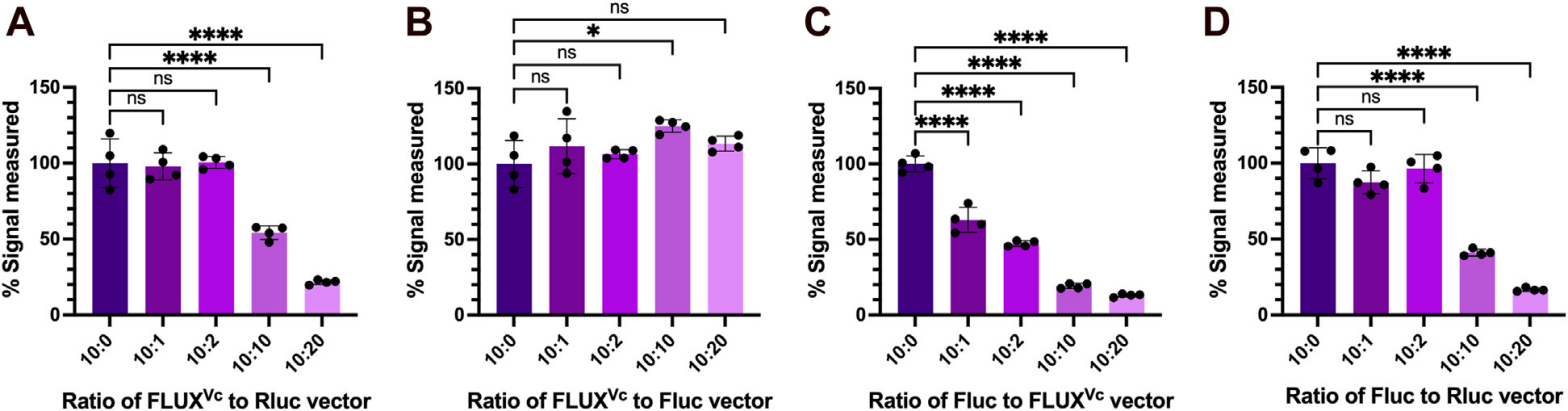
**Signals of the target luciferase measured at various ratios of target:control vectors.** Three types of luciferase vectors including, pGL3[luc+/SV40] vector, pGL3 [*FLUX*^*Vc*^/SV40], and pRL-TK vectors were used as either target vector or control vector for exploring the four-combination target/control vector sets including (*A*) FLUX^Vc^/Rluc, (*B*) FLUX^Vc^/Fluc, (*C*) Fluc/FLUX^Vc^, and (*D*) Fluc/RLuc. Constant amounts of target vectors (0.07 pmol) were cotransfected into HEK293T cells with varying amounts of control vector at ratios of 10:0, 10:1, 10:2, 10:10, and 10:20. Cells were collected at 48 h post-transfection and lysed in Passive Lysis Buffer (PLB) or Lux Lysis Reagent (LLR) and luciferase activity was independently measured. The activity of FLUX^Vc^ was monitored by adding 100 μl of an assay reagent cocktail consisting of 5 μM FMN, 100 μM HPA, 10 μM decanal, and 100 μM NADH in 50 mM sodium phosphate buffer pH 7.0 into a solution of cell lysate freshly mixed with 50 mU of reductase C_1_. The luminescence signal was monitored for 10 s with a 2 s delay using an AB-2250 single tube luminometer. The Fluc and Rluc activities were measured using firefly Luciferase Assay Reagent and *Renilla* Luciferase Assay Reagent, respectively according to the manufacturer’s instructions. Percentage of signals measured (% signal measured) was calculated by dividing the signals of the target luciferase with the signals obtained in the absence of control vector (designated as 10:0 ratio) and then multiplied by 100. Data are presented as mean ± SD of four biological replicates. ANOVA test was used to evaluate significance; ∗*p* < 0.05; ∗∗∗∗*p* < 0.0001; NS, not significant. FMN, flavin mononucleotide; HPA, *p*-hydroxyphenylacetic acid.

In transient transfection experiments, two types of vectors including target and control vectors are generally required, and the results shown in the previous section indicate that the FLUX^Vc^ system developed here gives signals suitable for reporter gene applications. We thus investigated the combined use of FLUX^Vc^ *versus* Fluc and Rluc in target/control reporter sets including (1) FLUX^Vc^/Rluc, (2) FLUX^Vc^/Fluc, (3) Fluc/FLUX^Vc^ in comparison with and (4) the standard Fluc/RLuc combination set generally used in luciferase bioreporter experiments. These combined vector sets with varying ratios of target:control vectors from 10:1 to 10:20 were used for measuring their light signals. Ideally, consistency of light signals with varying ratios of vectors would indicate robustness of a reporter system as a measurement tool. In real practical experiments, concentrations of target and control vectors may not be exactly the same at each transfection reaction and a good reporter tool should not be overly affected by this variation as this could create artifact signals that interfere with the effects of the experimental parameters one wants to address (55). In this experiment, we measured signals generated by target reporters with varying amounts of control reporters (Fig. S10) and compared these values with the target reporter signals in the absence of control vector (calculated as % signal measured, Fig. 6). Results in Figure 6 showed that the combination set 2. FLUX^Vc^/Fluc, showed the most consistent light signals when varying the vector ratios because their percent signal measured was almost unperturbed even at the highest amount of control vector (target:control = 10:20) (Fig. 6*B*). The use of FLUX^Vc^/Rluc is also acceptable for target:control vector ratios of up to 10:2. In the standard combination of Fluc/Rluc (Fig. 6*D*) and FLUX^Vc^/Rluc (Fig. 6*A*), the systems gave consistent percent signal measured with target:control ratios of 10:1 to 10:2 and the signals decreased at the lower target:control ratios. The behavior of the standard Fluc/Rluc combination was similar to those reported previously (55). Another set of combined use of Fluc/FLUX^Vc^ (Set 3) also gave high variation in percent signal measured when the target:control vector ratios were varied (Fig. 6*C*). Altogether, the data indicate that the combined use of FLUX^Vc^/Fluc (Set 2) gave the most ideal properties for their combined use as a target and control reporter set.

### Demonstrating the use of combined FLUX^Vc^/Fluc-reporter gene systems for investigating activators/inhibitors of the NF-κB cell signaling pathway

The NF-κB transcription factor plays a critical role in inflammation, immunity, and cell proliferation, differentiation, and survival which are relevant to several diseases including cancers (56, 57). Therefore, bioactive compounds capable of controlling signal transduction and gene regulation of this pathway are desired candidates for development of active pharmaceuticals (56, 58). Luciferase-reporter gene assays have been used as a valuable technique for screening NF-κB bioactive compounds because of their high sensitivity and broad range of detection. Therefore, we used the new combined FLUX^Vc^/Fluc-reporter system to measure the effects of TNFα, a known activator of the NF-κB signaling pathway, and compared it with the use of the standard Fluc/Rluc gene reporter combination.

**Figure 7:**
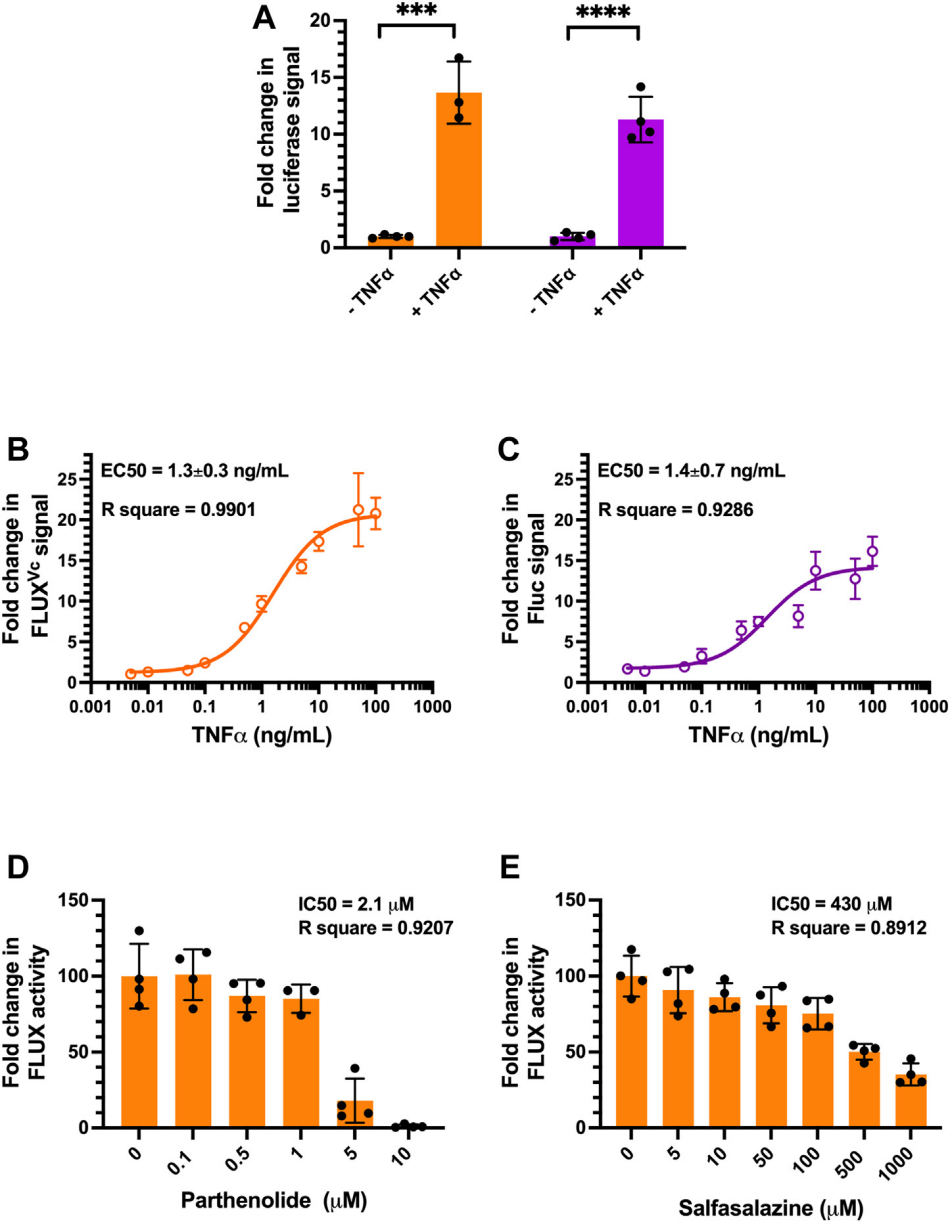
**Screening of inhibitors of the NF-κB cell signaling pathway using two combined luciferase-reporter gene assays.***A*, comparison of sensitivity response of two combined luciferase-reporter gene assays including (1) FLUX^Vc^/Fluc (*orange*) and (2) Fluc/Rluc (*purple*). Each combination including pGL3-NF-κB[*FLUX*^*Vc*^/TK] vector with pGL3 [*luc*^*+*^/SV40] vector (*orange*) and pGL3-NF-κB[*luc*^*+*^/TK] vector with pRL-TK vector (*purple*) were transfected into HEK293T cells. At 24 h after transfection, the old medium was changed to fresh medium either with 10 ng/ml of TNFα (+TNFα) or without TNFα (−TNFα) for 6 h. Cells were collected and lysed in either Passive Lysis Buffer (PLB) or Lux Lysis Reagent (LLR). The luciferase activity was then measured. Data are presented as mean ± SD of four biological replicates. Student's *t* test was used to evaluate significance; ∗∗∗*p* < 0.001; ∗∗∗∗*p* < 0.0001. *B* and *C*, investigation of TNFα dose-response of (*B*) FLUX^Vc^/Fluc and (*C*) Fluc/Rluc combinations. Experiments were carried out by transfecting HEK293T cells that were seeded in a 6-well plate for 1-day with (1) pGL3-NFκB[*FLUX*^*Vc*^/TK] with pGL3 [*luc*^*+*^/SV40] and (2) pGL3-NF-κB[*luc*^*+*^/TK] with pRL-TK vectors. After 12 h of transfection, transfected cells were washed, trypsinized, and seeded onto a 96-well plate. Seeded cells were further incubated for 24 h. Then, the old medium was changed to a new medium supplied with various TNFα activators for 6 h. Cells were collected and lysed in either Passive Lysis Buffer (PLB) or Lux Lysis Reagent (LLR) and their luciferase activities were measured. *D* and *E*, NF-κB inhibitor screening using the new combined *FLUX*^*Vc*^/*Fluc* reporter gene assay. The pGL3-NF-κB[*FLUX*^*Vc*^/TK] and pGL3 [*luc*^*+*^/SV40] vectors were transfected into HEK293T cells that were seeded in a 6-well plates for a 1-day period. After 12 h of transfection, transfected cells were washed, trypsinized and seeded onto a 96-well plate culture plate and further incubated for 24 h. The medium was then changed to fresh medium supplied with various concentrations of inhibitor for 30 min before activating the system by adding 10 ng/ml of TNFα for 6 h. The cells were collected and lysed in either Passive Lysis Buffer (PLB) or Lux Lysis Reagent (LLR), and their luciferase activities were measured. The activity of FLUX^Vc^ was monitored by adding 100 μl of a reagent cocktail consisting of 5 μM FMN, 100 μM HPA, 10 μM decanal, and 100 μM NADH in 50 mM sodium phosphate buffer pH 7.0 into cell lysate freshly mixed with 50 mU of C_1_ reductase. The luminescence signal was monitored for 10 s with a 2 s delay using an AB-2250 single tube luminometer. The Fluc and Rluc activities were measured using firefly luciferase and Renilla Luciferase Assay Reagents, respectively according to the manufacturer’s instructions. Data are presented as mean ± SD of four biological replicates. FMN, flavin mononucleotide; HPA, *p*-hydroxyphenylacetic acid; TNF, tumor necrosis factor.

To compare the sensitivity of both FLUX^Vc^/Fluc and Fluc/Rluc-reporter systems in measuring the effects of TNFα, the *FLUX*^*Vc*^ and *luc*+ reporter genes were constructed as reporter genes downstream of six tandem repeats of NF-κB transcriptional element with a thymidine kinase (TK) promotor to obtain the pGL3-NF-κB[FLUX^Vc^/TK] vector and a pGL3-NF-κB[*luc*^+^/TK] vector, respectively (Fig. S11, *A* and *B*). Combined sets of luciferase-reporter gene systems including (1) pGL3- NF-κB [*FLUX*^*Vc*^/TK] vector with pGL3 [*luc*^*+*^/SV40] vector and (2) pGL3[*luc+*/TK] vector with pRT-TK control vector were transfected into HEK293T cells in the presence or absence of TNFα. The results showed that both sets of vector combinations gave similar signal increment responses to TNFα, 14 ± 3-folds, and 11 ± 2-folds increment for FLUX^Vc^/Fluc and Fluc/Rluc combination, respectively (Fig. 7*A*, orange bar and purple bar, respectively). By varying concentrations of TNFα, the effects of TNFα dose-response on each reporter system can be calculated. The results showed sigmoidal dose-response curves corresponding to EC_50_ of TNFα of 1.3 ± 0.3 ng/ml and 1.4 ± 0.7 ng/ml for FLUX^Vc^/Fluc and Fluc/Rluc combination, respectively (Fig. 7, *B* and *C*, respectively). The data clearly showed that a new combination of FLUX^Vc^ as the target vector and Fluc as the control vector displays similar sensitivity to the standard combined Fluc/Rluc-reporter gene. It should be noted that the FLUX^Vc^/Fluc gave a slightly better and consistent curve, with a lower variation value, 1.3 ± 0.3 ng/ml *versus* 1.4 ± 0.7 ng/ml of the Fluc/Rluc reporter pair.

We further tested the ability of the new combined luciferase-reporter gene system for examining the inhibition of NF-κB using the known NF-κB inhibitors, parthenolide, and sulfasalazine (59, 60). The pGL3-NF-κB [*FLUX*^*Vc*^/TK] vector and pGL3[*luc+*/SV40] control vectors were transfected into HEK293T cells and the resulting transfected cells were incubated with various concentrations of the tested drugs. The results showed that IC_50_ of parthenolide and sulfasalazine were calculated as 2.1 μM and 430 μM, respectively (Fig. 7, *D* and *E*, respectively). These values are similar to those previously reported values, 1.5 μM and 625 μM for parthenolide and sulfasalazine, respectively (61, 62). Altogether, our results clearly indicate that the FLUX^Vc^/Fluc reporter system can serve as an alternative luciferase-reporter gene assay which gives good precision and robustness for screening of pharmaceutical active compounds such as inhibitors of the NF-κB pathway. The low cost of FLUX^Vc^ (∼1/100 or ∼1/150 compared to other gene reporters) would allow researchers to access technology without a high cost barrier, creating opportunities for life science development around the world.

### The lux activity is not sensitive to inhibition by polyphenol derivatives, allowing the use of the FLUX^Vc^ reporter gene for future screening of secondary plant metabolites

**Figure 8:**
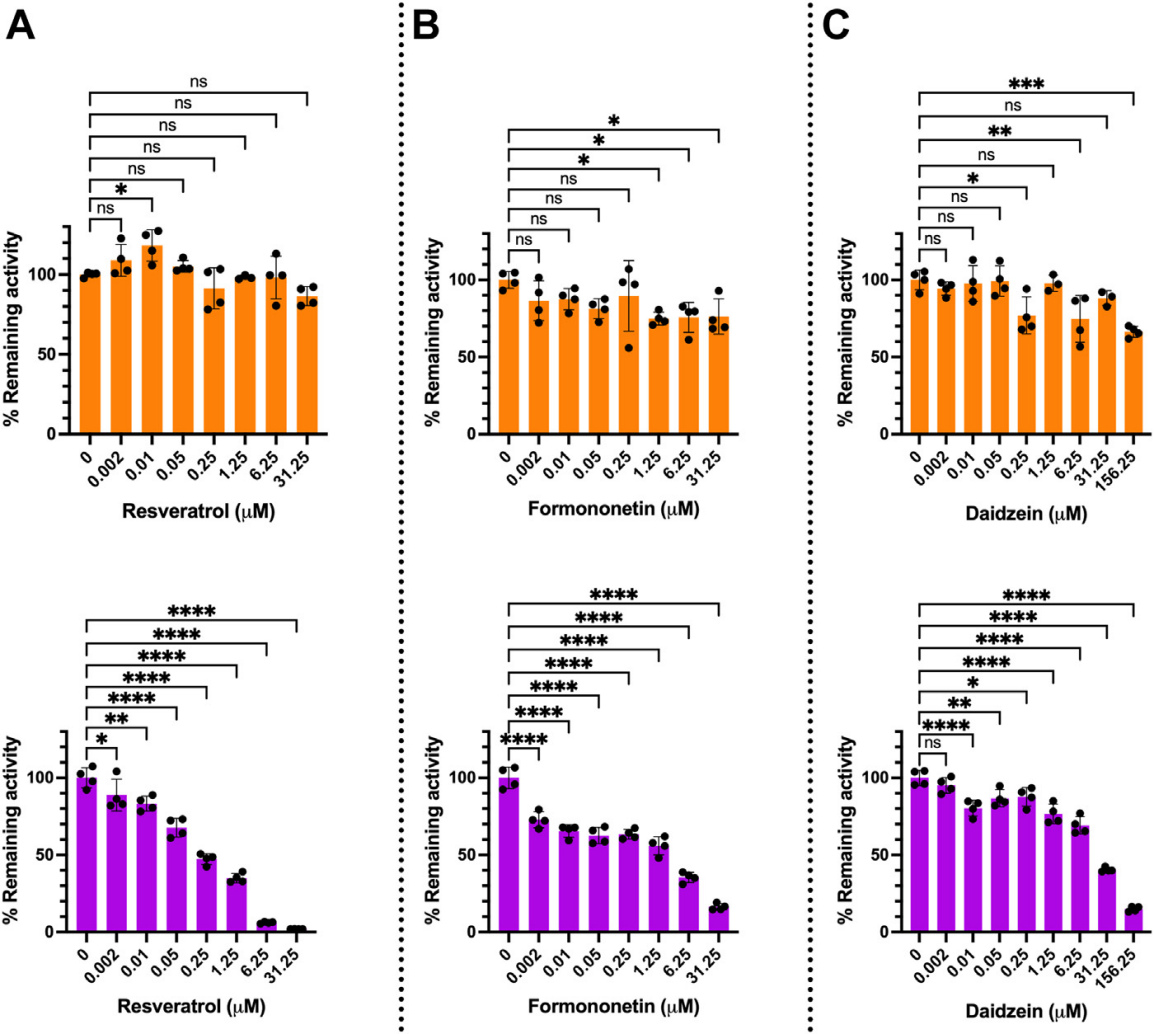
**Activities of purified Lux and Fluc in the presence of polyphenolic compounds.** Purified Lux (*orange*) or Fluc (*purple*) was incubated with polyphenolic compounds including (*A*) resveratrol, (*B*) formononetin, and (*C*) daidzein at various concentrations for 10 min before being taken out to measure their activities. The activity of Lux was monitored by adding an assay cocktail solution (100 μl) consisting of 5 μM FMN, 100 μM HPA, 10 μM decanal, and 100 μM NADH in 50 mM sodium phosphate buffer pH 7.0 into the Lux sample which was freshly mixed with 50 mU of the C_1_ reductase. While the activity of Fluc was monitored by adding an assay cocktail solution (100 μl) consisting of 100 μM D-luciferin, 2 mM ATP, 2 mM MgCl_2_, and 0.2 mM Coenzyme A in 50 mM 4-(2-hydroxyethyl)-1-piperazineethanesulfonic acid (HEPES) pH 7 into Fluc sample. The luminescence signal was monitored for 10 s with a 2 s delay using an AB-2250 single tube luminometer. Activities in the presence of polyphenols were divided by the activity measured in the absence of any polyphenol compounds to compare effects of the compounds; the values were multiplied by 100 to obtain % remaining activity. Data are presented as mean ± SD of four biological replicates. ANOVA test was used to evaluate significance; ∗*p* < 0.05; ∗*p* < 0.01; ∗∗∗*p* < 0.001; ∗∗∗∗*p* < 0.0001; NS, not significant. FMN, flavin mononucleotide; Fluc, firefly luciferase; HPA, *p*-hydroxyphenylacetic acid.

Polyphenol derivatives are among the bioactive compounds commonly investigated for their therapeutic use because of their wide biological activities (63) and due to their common availability from various higher plants including vegetables, fruits, cereals, legumes, herbs, and spices (64, 65). As it is known that some of these compounds can affect luciferase-based reporter assays (13, 66), we thus explored whether the Lux activity can be affected by these compounds. The effects of polyphenol compounds including resveratrol, daidzein, and formononetin (Fig. S12) on Lux and Fluc activities were investigated and compared. The results showed that these compounds do not affect Lux activities, allowing maintenance of nearly 100% Lux activity at all concentrations investigated (Fig. 8, *A*–*C*, orange). On the contrary, the Fluc activity is significantly affected by these compounds with 0.25 μM resveratrol, 6.25 μM formononetin, and 31.25 μM daidzein, decreasing the Fluc activity by 50% (Fig. 8, *A*–*C*, purple). The data suggest that these compounds, particularly resveratrol, are strong inhibitors of Fluc possibly due to their ability to bind to the enzyme because of their structural similarity to the native D-luciferin (Fig. S12) (12, 13). The ability of FLUX^Vc^ to withstand artifacts influenced by these compounds suggest that the FLUX^Vc^ reporter gene should be useful for screening the biological activities of plant polyphenols in the future.

### A general guideline for using *FLUX*^*Vc*^ as a gene reporter

A *FLUX*^*Vc*^ reporter gene consists of 2076 nucleotide bases encoding for 692 residues (Fig. S4) of the optimized *lux* gene for heterologous expression in mammalian cells. The complete *FLUX*^*Vc*^ sequence is also available in the National Center for Biotechnology Information database with GenBank number MZ393808. The *FLUX*^*Vc*^ gene can be used for constructing a reporter gene in any mammalian expression vector by placing it downstream of a constitutive/inductive promotor or transcription element. For transient transfection, either the *Fluc* gene or *Rluc* gene can be used as a control for the *FLUX*^*Vc*^ target vector. Transfection conditions and FLUX^Vc^ assay reagents are summarized in Table 1.

**Table 1:** Transfection conditions and assay reagents for FLUX^Vc^ reporter gene applications

Conditions/Reagents	Components	
Transfection ratio	10:1 of target: control vectors	
1X Lux Lysis reagents (LLR)	1% (w/v) CHAPS, 1 mM EDTA, 10% (w/v) glycerol in 50 mM sodium phosphate buffer pH 7.0.	
	Recommended volume of LLR for different types of multiwell plates (38).	
	**Multiwell plate size**	**1x LLR per well**
	24-well plate	100
	48-well plate	65
	96-well plate	20
	Experimental steps for cell lysis (38) 1. Remove all the medium. 2. Gently add 1x PBS buffer to wash the attached cells by swirling and removing the washing solution by pipetting the solution out. 3. Add LLR to lyse the treated cells. 4. Rock the culture plate at room temperature for 15 min. 5. Store the solution from (5.) at −80 °C freezer for further analysis.	
1x Lux assay reagents	5 μM flavin mononucleotide, 100 μM HPA, 100 μM NADH, and 10 μM decanal in 50 mM sodium phosphate buffer pH 7.0. Experimental steps for activity assays (38) 1. Add 2–10 μl of cell lysate and 50 mU of C_1_ reductase into 55-mm test tube. 2. Measure bioluminescence intensity by injecting 100 μl of 1x Lux assay reagent into the solution of cell lysate and C_1_ reductase. 3. Monitor reaction signals for 10 s with a 2 s delay time. 4. Integrate the peak area with a window *x*-axis of 10 s and report as a value of relative light unit (RLU).	

## Discussion

This report describes the construction, optimization, validation, and demonstration of applications of Lux from *V. campbellii* (*Vc*_Lux) for luciferase-reporter gene assays in mammalian cell systems. By changing the codon usage of the *Vc*_Lux gene and optimizing assay reagents, we created the Flavin Luciferase from *Vibrio campbllii* (*Vc*) for Mammalian Expression (FLUX^Vc^) system, which can be used as a gene reporter for mammalian cell expression. Based on detailed comparison of FLUX^Vc^ and the existing FLuc, FLUX^Vc^ shows higher S/N ratios than FLuc in HepG2 cells and comparable signals in other cell types. Intracellular FLUX^Vc^ is quite stable inside the cell for more than 72 h, suggesting that it can be used in HTS with high sensitivity over a broad detection range. We demonstrated that FLUX^Vc^ could be used as the target vector and control vector. The combination using FLUX^Vc^ as a target vector and Fluc as a control vector gave the most consistent signal output even better than using the combined Fluc and Rluc set. The new combined FLUX^Vc^/Fluc is a sensitive detection tool which can be used for detecting TNFα and for screening of inhibitors of the NF-κB cell signaling pathway.

The cost of FLUX^Vc^ assays is much lower than other systems. The combined FLUX^Vc^/Fluc system would allow the use of luciferase reporter genes with a much lower price than other systems. The price of the FLUX^Vc^ assay was calculated based on the costs of all substrates including decanal, FMN, HPA, NADH, and sodium phosphate buffer according to the price from Sigma-Aldrich. The cost of C_1_ reductase was also included based on the cost of in-house enzyme production. The current calculated price for Lux cocktail reagents is 0.01 United States dollar/assay, while the cost per assay of commercial firefly and Rluc based on Promega’s price is 1.22 and 1.70 United States dollar/assay, respectively.

The newly combined *FLUX*^*Vc*^*/Fluc* gene-reporter system gave the most consistent signals amongst all systems tested (Figs. 6*B* and S10*B*). It can give consistent signals throughout a wide range of target:control ratios (10:1 up to 10:20) which is broader than the commonly used Fluc/Rluc gene reporter system (Figs. 6*D* and S10*D*). The main reason behind this distinguishing feature is not clear. However, we hypothesize that this is due to the stability of intracellular half-life of FLUX^Vc^. The intracellular half-life of FLUX^Vc^ measured by inhibiting the translation process using cycloheximide was found to be much longer than 4 h (data not shown) which is significantly longer than Fluc and RLuc (3 and 4.5 h, respectively (7)). Because cells have limitations for exogenous expression of all transfected genes (67, 68), high amounts of control vector for transfection may affect protein translation of the target reporter gene as is the case for data shown in Figure 6, *A*, *C*, and *D*. The long intracellular half-life of FLUX^Vc^ would make the system stably express its gene with less sensitivity toward the amount of the control vector used. The stable signals of FLUX^Vc^ after the post-transfection period (Fig. 5*B*) also provides better advantages in transient transfection because a stable reporter gene gives less variation signal due to assay timing (69) and is suitable as a reporter system in HTS applications because the system would be able to tolerate the long period of time needed for processing thousands of samples. The potential application of FLUX^Vc^ in drug screening has been shown in measurement of IC_50_ of the known NF-κB inhibitors, parthenolide, and sulfasalazine (Fig. 7, *D* and *E*, respectively). However, the control vector of the FLUX^Vc^ reporter gene is not limited to only Fluc and Rluc. Researchers can employ a cheaper vector such as β-galactosidase pSV-β-Galactosidase as a control vector.

The *FLUX*^*Vc*^ reporter gene can be overexpressed in various types of cell line such as HEK293, NIH3T3, COS, HepG2. The first three cell lines are the most commonly used cell lines for biomedical research (70). In particular, HEK293 cells and its derivatives have been used extensively in the transfection-based experiments, protein expression, and productions of biologics and pharmaceuticals production (71). They have high efficiency of transfection and protein production and demonstrate reliable translation and processing of protein targets (72). Although the ability of the *FLUX*^*Vc*^ reporter gene to be overexpressed in HEK293T cells was less than that in COS1, HepG2, and NIH3T3 cells, the obtained signals are sufficient for detection of dynamic changes in cell signaling in response to effectors as illustrated by measuring TNFα effects on the NF-κB cell signaling pathway (Fig. 7). As our experiments demonstrated that the *FLUX*^*Vc*^ reporter gene can function well even in the least favorable cells, the results endorse the use of FLUX^Vc^ system in more favorable HepG2, COS1, NIH3T3 cells. Therefore, we think that the *FLUX*^*Vc*^ reporter gene should be a valuable tool for transfection-based experiments and their related implications such as protein tracking, promoter screening, cell signaling, and bioactive compounds screening. We believe that the *FLUX*^*Vc*^ reporter gene can be applied for detecting active compounds for other cell signaling pathways such as PI3K/Akt and JAK/STAT signaling pathways that regulate cell growth, proliferation, migration, and apoptosis, which are critical process for tumorigenesis that is a serious problems around the world (3, 73, 74). For screening bioactive compounds from plants such as resveratrol and benzothiazoles derivatives or other known inhibitors of Fluc (12, 75, 76), the FLUX^Vc^ showed a particular advantage over the Fluc system because the FLUX^Vc^ enzyme activity is not sensitive to inhibition by these compounds, allowing their biological effects on promoters to be directly assessed without artificial influence.

We also noted that the *FLUX*^*Vc*^ reporter gene gives higher S/N than that the Fluc reporter gene in HepG2 cells (Fig. 4). HepG2 is a human hepatocellular carcinoma cell which has drug metabolizing enzymes comparable to normal hepatocytes. The cells can be cultivated *in vitro* and used for drug testing conveniently (77). Although HepG2 is less used for investigating cellular signaling and events than other cell types, it is commonly used for drug cytotoxicity screening and investigating drug toxic mechanisms. We hope that the *FLUX*^*Vc*^-reporter gene developed here which can be expressed well in HepG2 cells can contribute to future drug cytotoxicity screening and many other applications related to this cell line.

## Experimental procedures

### All chemical, cell cultures, strains, and vectors

All laboratory reagents used were analytical grade and mostly purchased from Sigma-Aldrich, Merck-Millipore, and Tokyo Chemical Industry. The *E. coli* XL1 (blue) and *E. coli* BL21 (DE3) were purchased from Merck-Millipore. Human Embryonic Kidney (HEK293T cells, RCB2202), Human hepatocyte carcinoma cancer cells (HepG2, RCB1886), Mouse embryonic fibroblast cells (NIH3T3 cells, RCB2767), and Monkey kidney cells (COS1 cells, RCB0143) were ordered from RIKEN BioResource Research. Molecular biology reagents, restriction endonucleases, T4 DNA ligase, and Gibson Assembly Master Mix were purchased from New England Biolabs. High-fidelity *Phusion* DNA polymerase was purchased from Thermo Fisher Scientific. Anti-fusion LuxAB (rabbit polyclonal) IgG was self-made while anti-beta-actin(C4) IgG (mouse monoclonal), mouse anti-rabbit IgG-horseradish peroxidase (HRP), and goat anti-mouse IgG-HRP were purchased from Santa Cruz Biotechnology. All culture medium, trypsin, penicillin-streptomycin, and FBS were purchased from Fujifilm Wako Pure Chemical Corporation, Luciferase assay system (E1500) and Rluc assay system (E2810) were purchased from Promega Corporation. The FlavoPrep plasmid DNA extraction mini kit and FlavoPrep gel/PCR purification kit were purchased from Flavogen Biotech corp. The oligonucleotide primers were synthesized by Integrated DNA Technologies. The DNA sequences were analyzed by an automated DNA sequencing machine service of A T C G Co, Ltd Lux and C_1_ reductase were purified according to described procedures (16, 78, 79).

### Mammalian cell culture, transient transfection, and cell harvesting

Human Embryotic Kidney (HEK293T) cells and human hepatocyte carcinoma cancer cells (HepG2 cells) were grown in Dulbecco’s Modified Eagle Medium (DMEM)–low glucose supplemented with 10% (v/v) heat-inactivated FBS plus 1% (w/v) penicillin-streptomycin. Mouse embryonic fibroblast cells (NIH3T3 cells) and monkey kidney cells (COS1) were grown in DMEM-high glucose supplemented with 10% (v/v) heat-inactivated FBS plus 1% (w/v) penicillin-streptomycin. Four types of cells were maintained at 37 °C with 5% CO_2_. For transient transfection, 1 × 10^5^ cells per well were plated and cultured in suitable DMEM media supplemented with 10% (v/v) heat-inactivated FBS in 24-well plates for 1 day prior to transfection. Cells were transfected by adding 0.07 pmol of target vector and 0.007 pmol of for control vector with lipofectamine3000 in DMEM-free serum. The transfected cells were maintained for 24 to 72 h. Cells were harvested by washing cells with 500 μl PBS buffer pH 7.4 and detached cells were collected using specific lysis buffer. A 1x PLB was used for collecting Fluc transfected cell while a 1x LLR was used for collecting FLUX^Vc^ transfected cells. The lysate was kept at −80 °C until used.

### Protein extraction and protein concentration determination

Cells were lysed by a freezing-thawing process (freezing at −80 °C for 10 min and thawing at room temperature water for 2 min). The cell lysate was collected by centrifugation at 12,000 rpm at 4 °C for 10 min. Total protein concentration was determined using Proteostain-Protein Quantification Rapid-Kit (Donjindo Molecular Technologies) according to the manufacturer’s instructions. The absorbance change at 595 nm was measured using a microplate reader spectrophotometer (iMarkTM, Bio-Rad). A plot of absorbance change *versus* BSA concentration was used as a standard curve. Protein concentrations of cell lysate were calculated based on the standard curve.

### Measurement of luciferase activity in cell lysate

Activity of luciferase was assayed by monitoring light emission using AB-2250 single tube luminometer (ATTO Corporation). The Lux cocktail reagent consisting of 5 μM FMN, 10 μM decanal, 100 μM HPA, and 100 μM NADH in 50 mM sodium phosphate buffer pH 7.0. was injected into a reaction chamber containing 2 to 10 μl of cell lysate and 50 mU of C_1_ reductase. The total volume of the assay was ∼110 μl. The light emission was monitored for 10 s with 2 s delay. Fluc and Rluc activities were measured using Fluc and Rluc Assay Reagents, E1500 and E2810 (Promega Corporation), respectively according to the manufacturer’s instructions. The integrated peak area was reported as relative light units. Signal of luciferase-based experiments were normalized using light emitted from the control vector.

### Measurement of purified Lux activity

Activity of purified Lux was assayed by monitoring light emission using an AB-2250 single tube luminometer (ATTO Corporation). A Lux cocktail reagent consisting of 5 μM FMN, 10 μM decanal, 100 μM HPA, and 100 μM NADH in 50 mM sodium phosphate buffer pH 7.0 was injected into a reaction chamber consisting of 2 μl of purified Lux and 50 mU of C_1_ reductase. The light emission was monitored for 10 s with 2 s delay. The integrated peak area was reported as relative light units.

### Western blot analysis

The cell lysate was separated by 12.5% (w/v) SDS-PAGE electrophoresis (ATTO Cooperation) and transferred onto a polyvinylidene difluoride membrane (Bio-rad). The membrane blots were blocked in 1x EzBlockChemi (ATTO Cooperation) for 1 h at room temperature and then incubated with primary antibody (Anti-fusion LuxAB IgG/Anti-β-actin IgG) which was diluted in Solution I of Western blot Immuno Booster (ATTO Cooperation) for 24 h at 4 °C. The membrane was washed by Tris Buffered Saline with Tween20 for three times before incubating in HRP-conjugated secondary antibodies (mouse-anti-rabbit HRP/goat-anti-mouse HRP) for 1 h at room temperature. The membrane was washed three times using Tris Buffered Saline with Tween20 before incubating in chemiluminescent reagent for HRP (EzWestLumi plus, ATTO Cooperation). The molecular weight marker bands were marked on the polyvinylidene difluoride membrane by pencil and was captured by WSE-6100 LuminoGraph I Gel documentation system (ATTO Cooperation). Protein staining using HRP-conjugated secondary antibodies was performed and bands were detected using a WSE-6100 LuminoGraph I Gel documentation system (ATTO Cooperation) using chemiluminescence mode.

### Validation of novel dual-luciferase assay using NF-κB transcription element

Four sets of validated reporter genes (NF-κB reporter vector/Internal control vector) including (1) pGL3-NF-κB[*luc*^+^/TK] vector/pRL-TK vector, (2) pGL3-NF-κB[*FLUX*^*Vc*^/TK] vector/pRL-TK vector, (3) pGL3-NF-κB[*luc*^+^/TK] vector/pGL3 [*FLUX*^*Vc*^/SV40] vector, and (4) pGL3-NF-κB[*FLUX*^*V*c^/TK]vector/pGL3[*luc+*/SV40] vector were independently transfected into 5 × 10^5^ HEK293T cells that were plated 1 day prior to transfection in 6-well plates using lipofectamine3000 in DEME-free serum. After 12 h post-transfection, the transfected cells were washed, trypsinized, and seeded on a 96-well plate. The culture plate was incubated for 24 h at 37 °C with 5% CO_2_. The culture medium was then changed to a new medium either supplied with 0.005 to 10 ng/ml of TNFα or without TNFα. The transfected cells were continuously stimulated for 6 h. Cells were harvested by washing cells with 500 μl PBS buffer pH 7.4 and collected using specific lysis regent. A 20 to 50 μl of 1x PLB was used for collecting Fluc transfected cell while 20 to 50 μl of 1x LLR was used for collecting bacterial luciferase transfected cells. The luciferase activity was independently measured according to the measurement protocol described above.

### Use of a new combined FLUX^Vc^/Fluc for demonstrating investigation of inhibitors of the NF-κB signaling pathway

The pGL3-NF-κB[*FLUX*^*Vc*^/TK] and pGL3 [*luc+*/SV40] vectors were cotransfected into 5 × 10^5^ HEK293T cells that were plated 1 day prior to transfection in 6-well plates using lipofectamine3000 in DEME-free serum. After 12 h post-transfection, the transfected cells were washed, trypsinized, and seeded on a 96-well plate. The culture plate was incubated for 24 h at 37 °C with 5% CO_2_. The culture medium was then changed to a new medium supplied with various concentrations of inhibitors for 30 min before stimulating the system by adding 10 ng/ml of TNFα for 6 h. The cells were then collected by adding 50 μl of LLR and luciferase activities were independently measured. The activity of Lux was monitored by adding 100 μl of a cocktail reagent consisting of 5 μM FMN, 100 μM HPA, 10 μM decanal, and 100 μM NADH into 2 to 10 μl of cell lysate freshly mixed with 50 mU of C_1_ reductase. The luminescence signal was monitored for 10 s with a 2 s delay using an AB-2250 single luminometer (ATTO Corporation). The Fluc activity was measured using Fluc Assay Reagent [E2810, Promega Corporation] according to the manufacturer’s instructions.

## Data availability

All data are contained in the article or Supporting Information.

## Supporting information

This article contains supporting information.

## Conflict of interest

The authors declare no conflict of interest.
