# Supplementary material for: High sensitivity and low-cost flavin luciferase (FLUXVc)-based reporter gene for mammalian cell expression
Source: J Biol Chem. 2023 Mar 24;299(5):104639. doi: 10.1016/j.jbc.2023.104639 (PMC10164909; doi:10.1016/j.jbc.2023.104639)
Supplement: Supporting Figures S1–S12 [file mmc1.docx]

**Supporting information for**

**High Sensitivity and Low-Cost Flavin luciferase (FLUX^Vc^)-based Reporter Gene for Mammalian Cell Expression**

Jittima Phonbuppha^1^, Ruchanok Tinikul^2^, Yoshihiro Ohmiya^3, 4^ and Pimchai Chaiyen^1, *^

Materials including Supplementary Figures 1-12

**
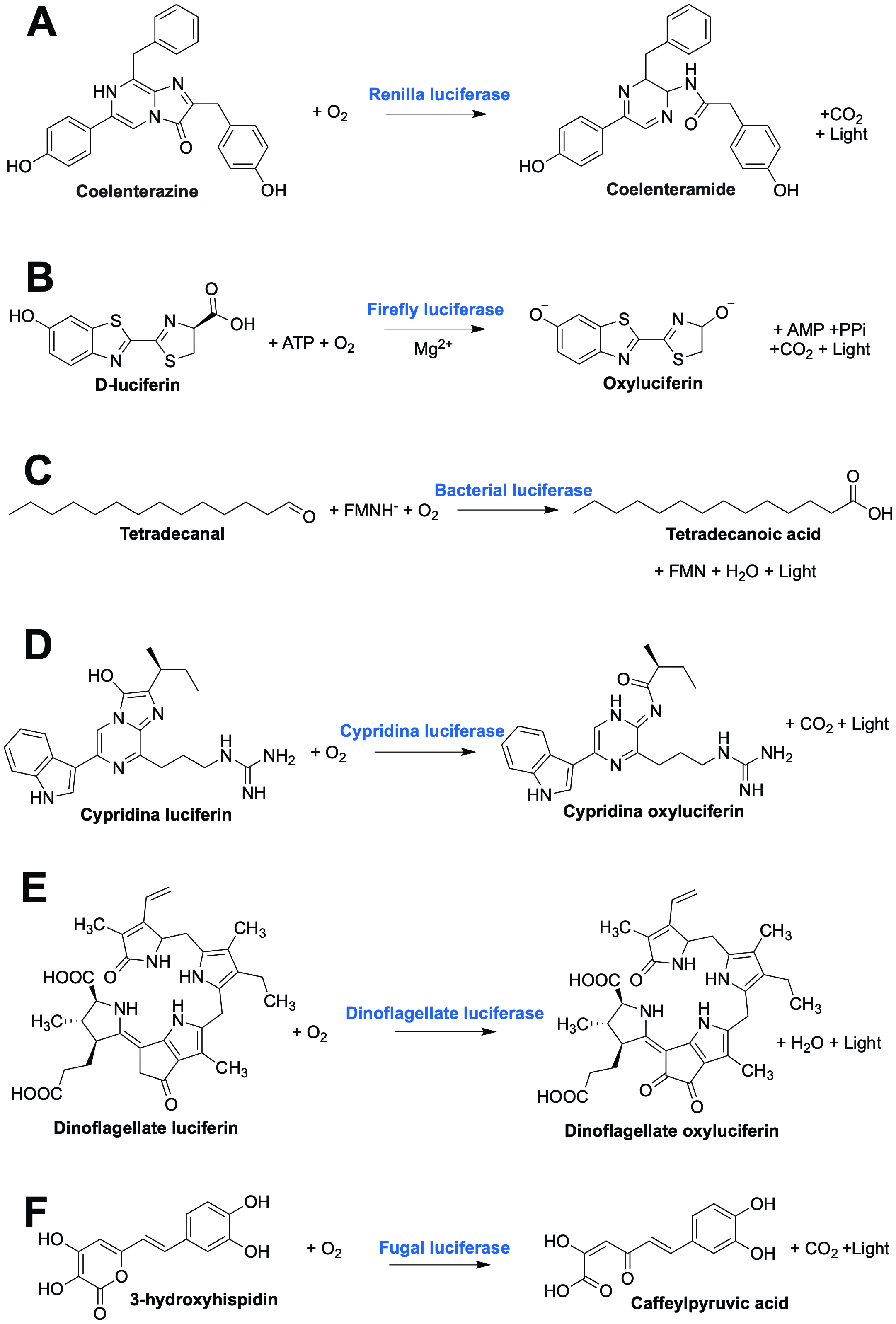
**

**Figure S1** Enzymatic reaction of six luciferases including **A.** Renilla luciferase **B.** firefly luciferase **C.** bacterial luciferase **D.** Cypridina luciferase **E.** Dinoflagellate luciferase and **F.** fugal luciferase.


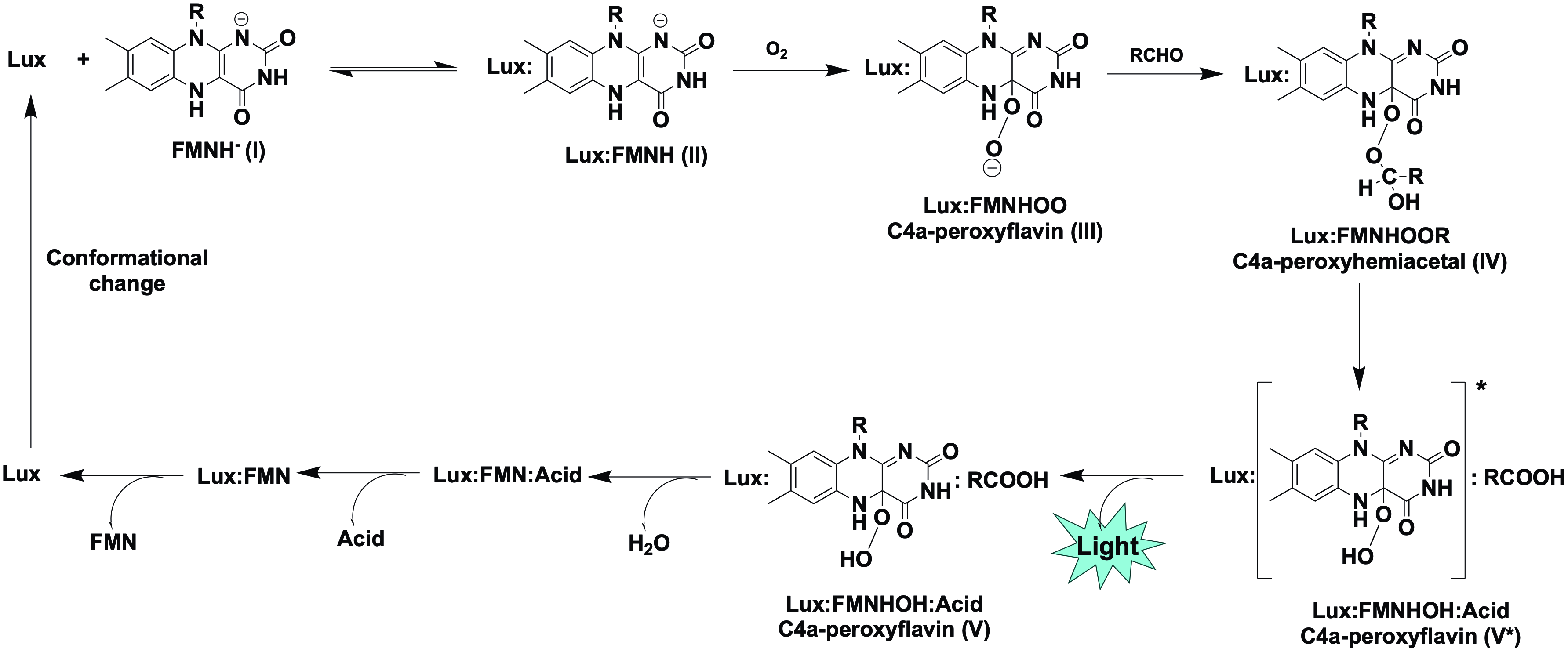


**Figure S2** Kinetic mechanisms of bacterial luciferase (Lux)


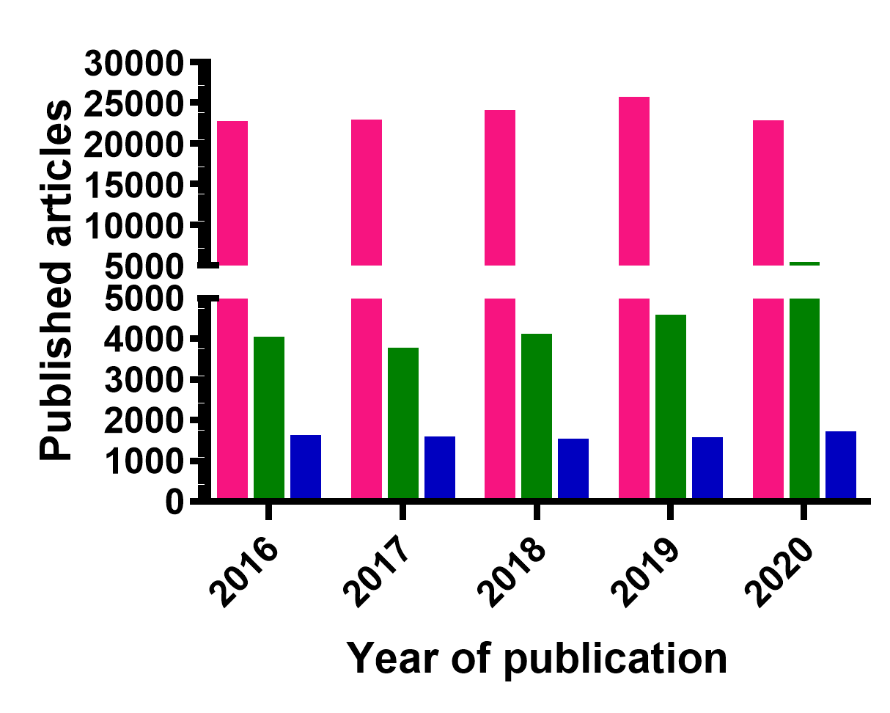


**Figure S3.** Published articles using all types of luciferase reporters (pink bar), the pGL3 vector (green bar) and the pGL4 vector (blue bar). Keywords containing either luciferase reporter, pGL3 or pGL4 were searched in google scholar to find related articles during 2016-2020.

1 ATGAAGTTTGGCAATTTCCTGCTGACATACCAGCCTCCTGAACTGAGCCAGACCGAAGTG 60

1 M K F G N F L L T Y Q P P E L S Q T E V 20

61 ATGAAGAGACTGGTGAACCTGGGCAAGGCCTCTGAGGGGTGCGGATTCGACACAGTGTGG 120

21 M K R L V N L G K A S E G C G F D T V W 40

121 CTGCTGGAACACCACTTCACCGAGTTCGGACTGCTGGGAAACCCATACGTGGCAGCTGCA 180

41 L L E H H F T E F G L L G N P Y V A A A 60

181 CACCTGCTGGGAGCAACTGAAAAGCTGAATGTGGGAACCGCCGCTATCGTCCTGCCCACA 240

61 H L L G A T E K L N V G T A A I V L P T 80

241 GCTCATCCTGTGAGGCAGGCAGAGGACGTGAACCTGCTGGATCAGATGTCAAAAGGCAGG 300

81 A H P V R Q A E D V N L L D Q M S K G R 100

301 TTCCGCTTTGGGATTTGCCGCGGACTGTATGACAAGGATTTTCGAGTGTTCGGGACCGAC 360

101 F R F G I C R G L Y D K D F R V F G T D 120

361 ATGGATAATAGCCGGGCCCTGATGGACTGTTGGTACGATCTGATGAAGGAAGGCTTTAAC 420

121 M D N S R A L M D C W Y D L M K E G F N 140

421 GAGGGGTATATCGCAGCCGACAATGAGCACATCAAGTTCCCTAAAATTCAGCTGAACCCA 480

141 E G Y I A A D N E H I K F P K I Q L N P 160

481 TCCGCCTACACACAGGGAGGGGCTCCAGTGTATGTGGTCGCCGAATCTGCTAGTACCACA 540

161 S A Y T Q G G A P V Y V V A E S A S T T 180

541 GAATGGGCTGCAGAGAGAGGACTGCCCATGATCCTGTCTTGGATCATTAACACACATGAG 600

181 E W A A E R G L P M I L S W I I N T H E 200

601 AAGAAAGCCCAGCTGGATCTGTACAATGAAGTGGCTACTGAGCACGGCTATGACGTGACC 660

201 K K A Q L D L Y N E V A T E H G Y D V T 220

661 AAAATCGATCATTGCCTGTCCTACATTACTTCTGTGGACCACGATAGCAACAAGGCTAAA 720

221 K I D H C L S Y I T S V D H D S N K A K 240

721 GACATCTGTAGGAATTTTCTGGGGCATTGGTACGATAGTTATGTGAACGCCACCAAGATT 780

241 D I C R N F L G H W Y D S Y V N A T K I 260

781 TTCGACGATTCAGACCAGACAAAGGGATATGACTTCAACAAGGGCCAGTGGCGCGACTTC 840

261 F D D S D Q T K G Y D F N K G Q W R D F 280

841 GTGCTGAAGGGCCACAAAGACACTAACCGGAGAATCGATTACAGCTATGAGATTAATCCA 900

281 V L K G H K D T N R R I D Y S Y E I N P 300

901 GTGGGAACCCCCGAGGAATGTATCGCTATCATTCAGCAGGACATTGATGCAACAGGCATC 960

301 V G T P E E C I A I I Q Q D I D A T G I 320

961 AACAATATTTGCTGTGGGTTTGAAGCAAACGGCAGCGAGGAAGAGATCATTGCCTCTATG 1020

321 N N I C C G F E A N G S E E E I I A S M 340

1021 AAGCTGTTCCAGAGTGACGTGATGCCTTACCTGAAGGAGAAACAGGTCATCAATATTTTT 1080

341 K L F Q S D V M P Y L K E K Q V I N I F 360

1081 GAAAAGGAGAGGGATCAGAAATTTGGCCTGTTCTTTCTGAACTTCATGAATAGTAAACGC 1140

361 E K E R D Q K F G L F F L N F M N S K R 380

1141 AGCTCCGACCAGATCATTGAAGAGATGCTGGATACAGCTCACTACGTGGACCAGCTGAAG 1200

381 S S D Q I I E E M L D T A H Y V D Q L K 400

1201 TTTGATACTCTGGCCGTCTATGAGAACCATTTCTCCAACAATGGAGTGGTCGGCGCCCCA 1260

401 F D T L A V Y E N H F S N N G V V G A P 420

1261 CTGACTGTGGCTGGATTCCTGCTGGGCATGACCAAGAACGCAAAAGTGGCCTCTCTGAAT 1320

421 L T V A G F L L G M T K N A K V A S L N 440

1321 CACGTCATCACTACCCACCATCCCGTGCGGGTCGCTGAAGAGGCATGCCTGCTGGACCAG 1380

441 H V I T T H H P V R V A E E A C L L D Q 460

1381 ATGAGTGAAGGCAGATTCGTGTTTGGGTTCAGTGACTGTGAGAAGTCAGCCGATATGCGA 1440

461 M S E G R F V F G F S D C E K S A D M R 480

1441 TTCTTTAACCGGCCCACCGATTCACAGTTTCAGCTGTTCAGCGAGTGCCACAAAATCATT 1500

481 F F N R P T D S Q F Q L F S E C H K I I 500

1501 AATGACGCCTTTACAACTGGCTACTGTCATCCTAACAATGACTTCTACAGCTTCCCCAAG 1560

501 N D A F T T G Y C H P N N D F Y S F P K 520

1561 ATCTCCGTGAACCCTCACGCCTTTACCGAGGGAGGCCCTGCACAGTTCGTCAATGCCACA 1620

521 I S V N P H A F T E G G P A Q F V N A T 540

1621 AGCAAGGAAGTGGTCGAGTGGGCCGCTAAACTGGGCCTGCCACTGGTGTTCAAGTGGGAC 1680

541 S K E V V E W A A K L G L P L V F K W D 560

1681 GATTCCAATGCACAGCGAAAAGAATACGCCGGCCTGTATCACGAGGTGGCACAGGCCCAC 1740

561 D S N A Q R K E Y A G L Y H E V A Q A H 580

1741 GGCGTGGACGTGAGCCAGGTCCGCCATAAGCTGACTCTGCTGGTGAACCAGAATGTCGAT 1800

581 G V D V S Q V R H K L T L L V N Q N V D 600

1801 GGCGAAGCAGCCAGAGCTGAGGCAAGGGTGTACCTGGAAGAGTTTGTCCGGGAAAGCTAT 1860

601 G E A A R A E A R V Y L E E F V R E S Y 620

1861 CCAAACACCGACTTCGAGCAGAAAATGGTGGAACTGCTGTCCGAGAATGCTATCGGGACC 1920

621 P N T D F E Q K M V E L L S E N A I G T 640

1921 TACGAAGAGTCTACACAGGCTGCAAGAGTGGCCATTGAGTGCTGTGGAGCCGCTGACCTG 1980

641 Y E E S T Q A A R V A I E C C G A A D L 660

1981 CTGATGTCATTCGAAAGCATGGAGGATAAGGCACAGCAGAGAGCCGTGATTGATGTCGTG 2040

661 L M S F E S M E D K A Q Q R A V I D V V 680

2041 AACGCAAATATCGTGAAATACCACTCATAA 2070

681 N A N I V K Y H S * 690

**Figure S4.** Nucleotide (2070 base pairs) and deduced amino acid (690 residues) sequences of the *FLUX^Vc^* gene. Highlighted in yellow is an artificial peptide linker which joins the *luxA* and *luxB* genes.


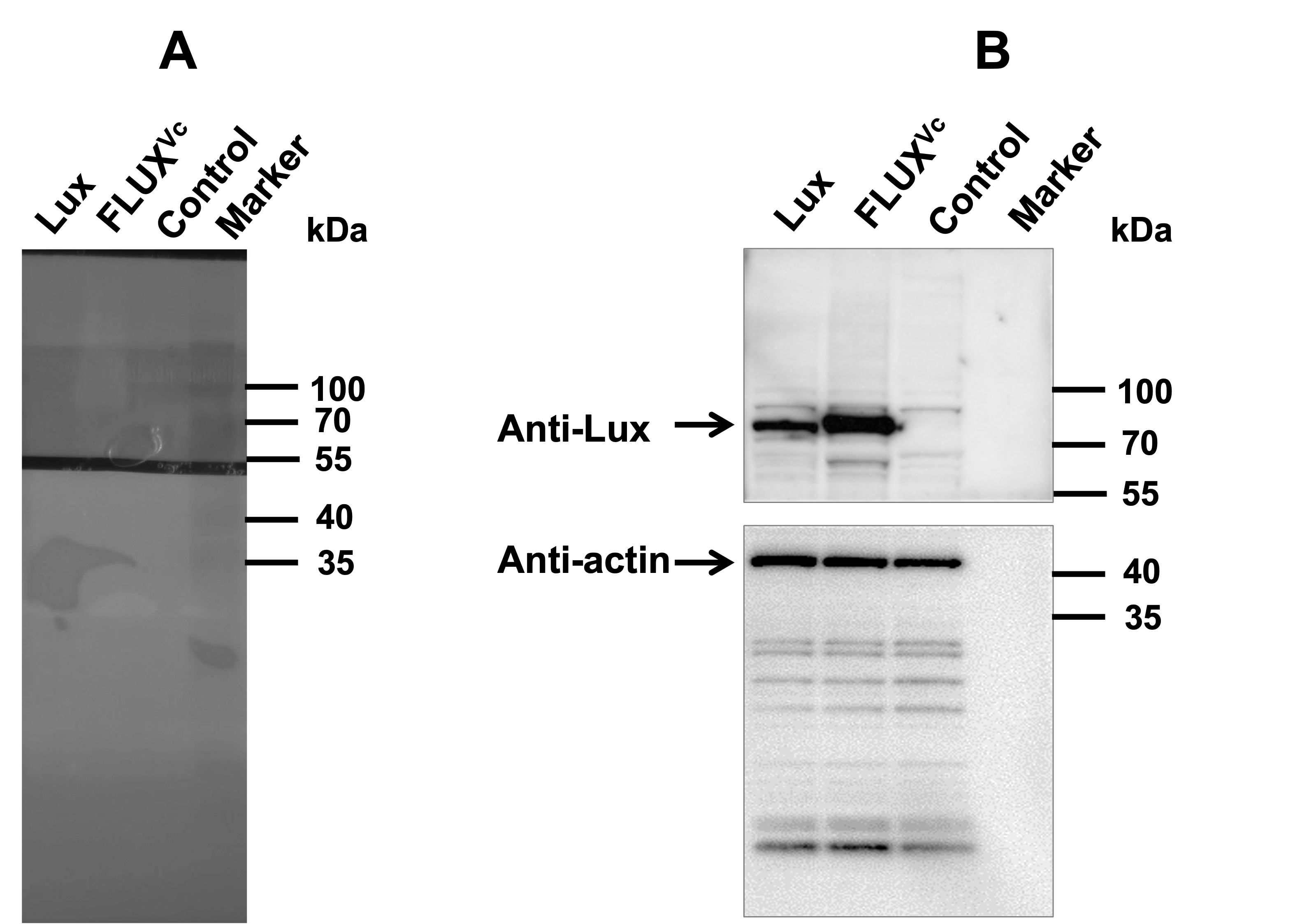


**Figure S5** Transferred membrane images which were captured in **A.** bright field mode and **B.** chemiluminescence mode. Protein markers were observed by eye and their positions were marked before capturing the image. Chemiluminescence mode was then used to detect antibody staining of LuxAB using the HRP-conjugated secondary antibody which specifically binds to anti-fusion LuxAB IgG or Anti-β-actin IgG. Note that B. is the same as shown in figure 1B (main text). The figure S5B here shows the complete gel of figure 1B (main text) to illustrate the detection of all proteins and protein markers using bright filed and anti-fusion LuxAB and anti-actin using chemiluminescence mode.

**
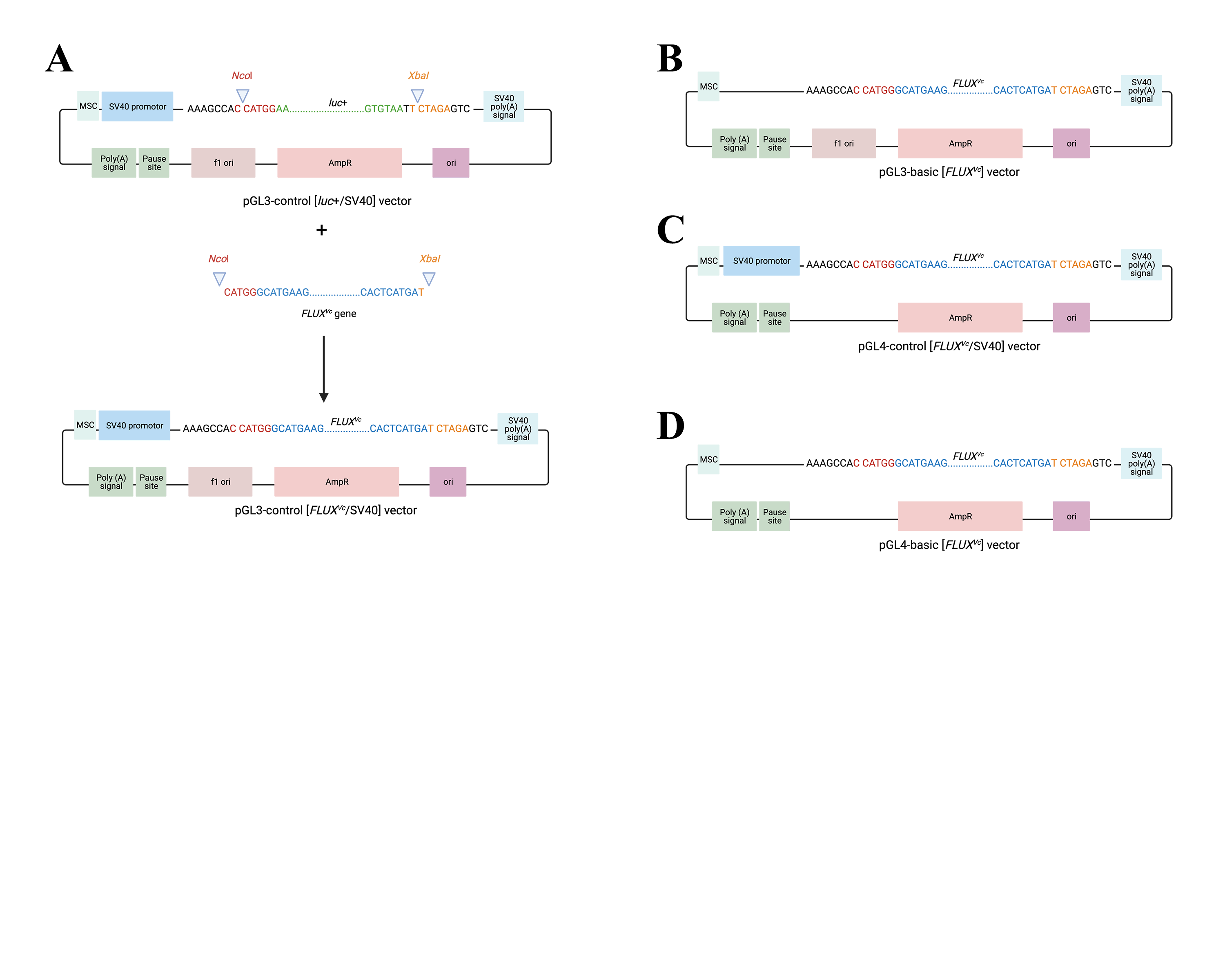
**

**Figure S6.** Maps of *FLUX^Vc^* genes constructed in two types of pGL vectors in the presence and absence of a constitutive SV40 promoter. **A.** The pGL3 [*FLUX^Vc^*/SV40] vector **B.** The pGL3 [*FLUX^Vc^*] vector **C.** The pGL4 [*FLUX^Vc^*/SV40] vector and **D.** The pGL4 [*FLUX^Vc^*] vector. The construction processes of each vector were all similar except for the pGL4 [*FLUX^Vc^*/SV40] vector. First, the pGL vector containing the Fluc gene was digested with *Nco*I and *Xba*I restriction enzymes to remove the *Fluc* gene. The *FLUX^Vc^* gene was amplified and digested by *Nco*I and *Xba*I restriction enzymes. The digested pGL vector was then ligated with the digested *FLUX^Vc^* gene to yield the pGL vector consisting of *FLUX^Vc^* as a reporter gene.

**
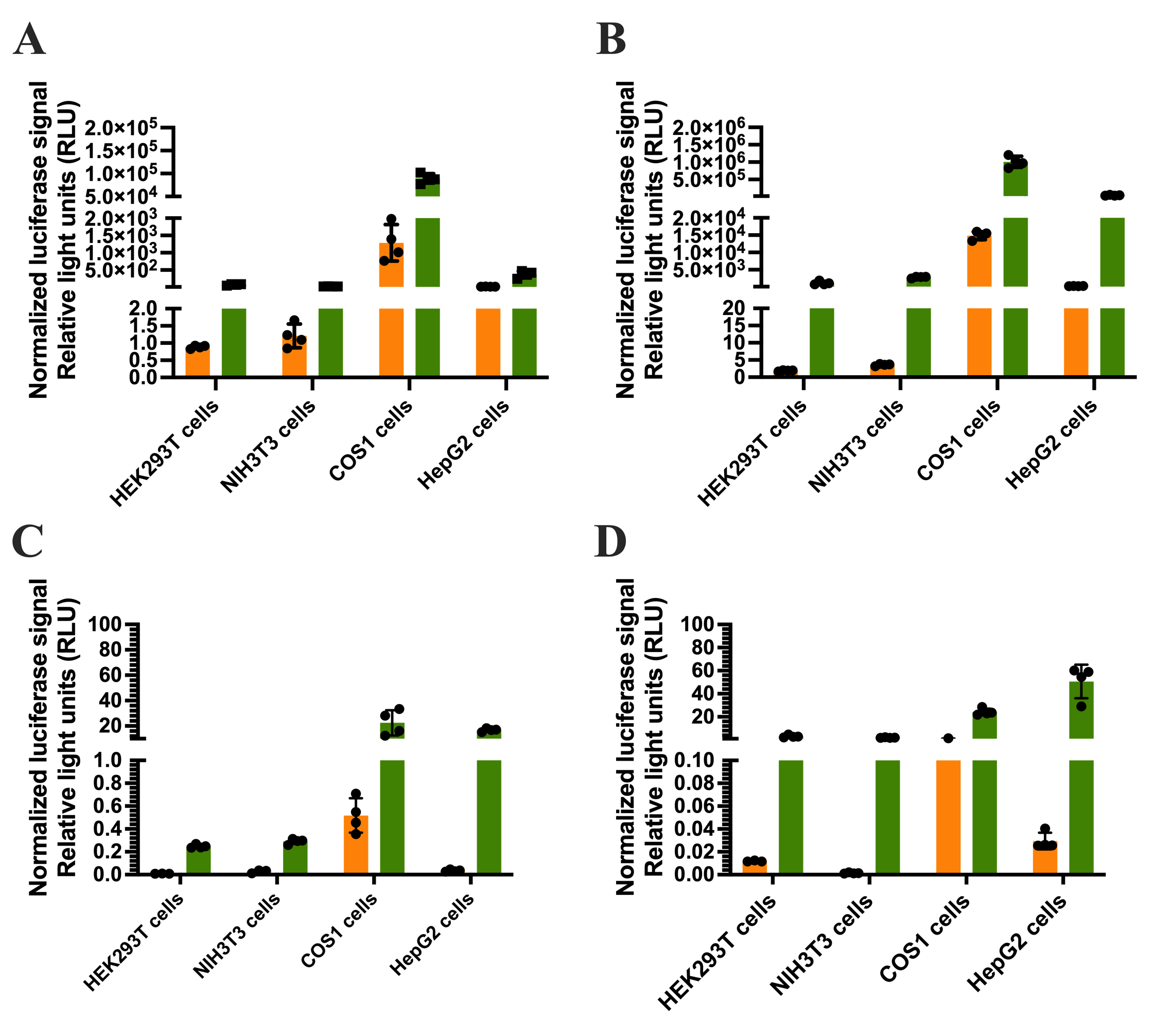
**

**Figure S7.** Comparison of normalized luciferase signals between FLUX^Vc^ (orange bar) and Fluc (green bar) in various cell types including HEK293T, NIH3T3, COS1 and HepG2 cells. **A.** pGL3 backbone vectors in the presence or **C.** absence of a constitutive SV40 promotor. **B.** pGL4 backbone vectors in the presence or **D.** absence of a constitutive SV40 promotor. Each reporter gene was co-transfected with the pRL-TK vector as an internal control for each cell type. Cells were collected at 48 h post-transfection in either Passive Lysis Buffer (PLB) or Lux Lysis Reagent (LLR) and luciferase activities were measured. The activity of FLUX^Vc^ was monitored by adding 100 µL of a cocktail reagent consisting of 5 µM FMN, 100 µM HPA, 10 µM decanal and 100 µM NADH in 50 mM sodium phosphate pH 7.0 into a cell lysate which was freshly mixed with 50 mU of C_1_ reductase. The luminescence signal was monitored for 10 sec with a 2 sec delay using an AB-2250 single tube luminometer. The Fluc and Rluc activities were measured using firefly luciferase and *Renilla* Luciferase Assay Reagents, respectively according to manufacturer’s instructions. The luciferase activity under the constitutive SV40 promoter was divided by their Rluc luciferase control activity to yield the normalized luciferase signals. Data are presented as mean±SD of four biological replicates.

**
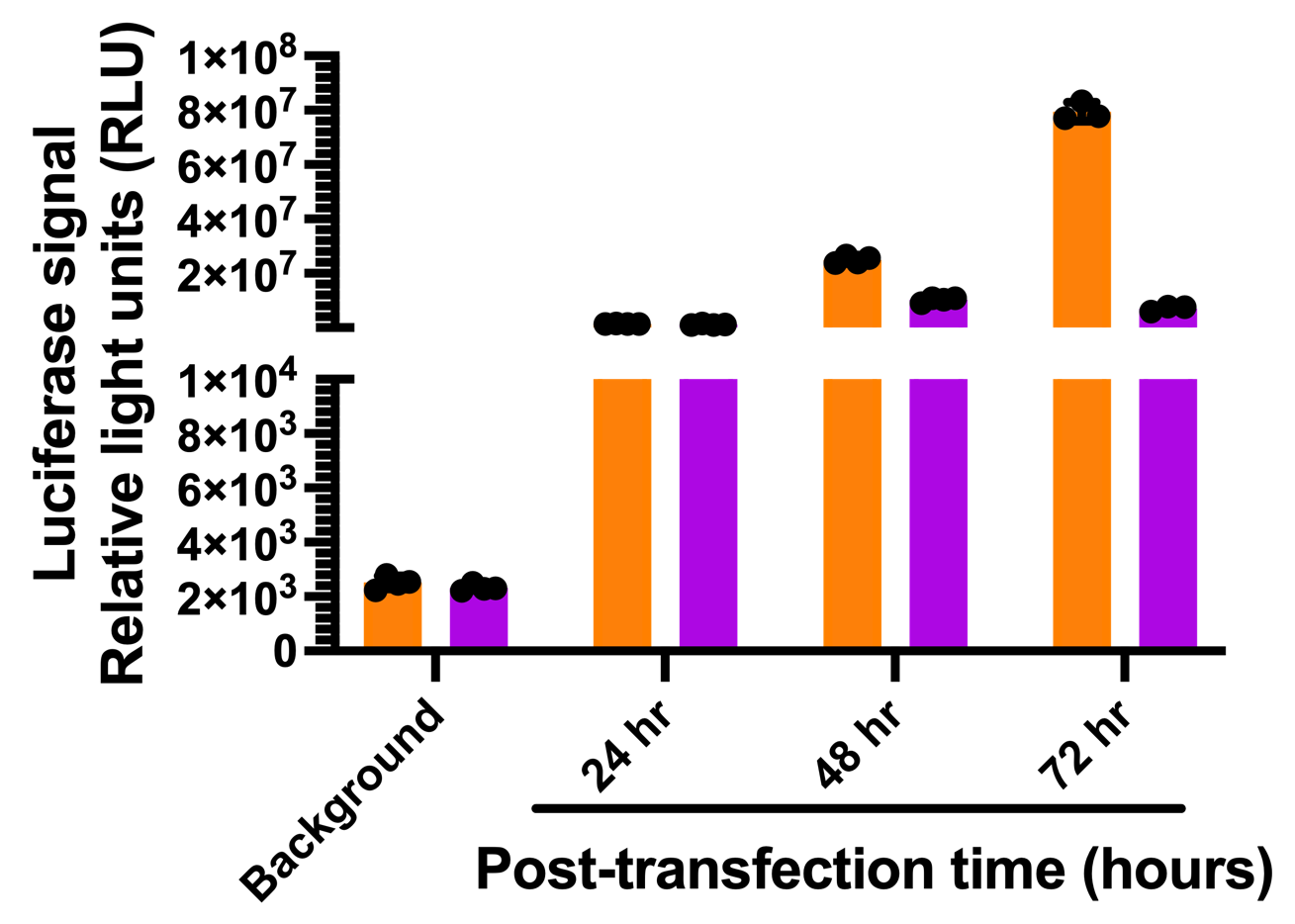
**

**Figure S8.** Effects of post-transfection period on FLUX^Vc^ signals. The pGL3 [*FLUX^Vc^*/SV40] vector (orange) was co-transfected with the pRL-TK vector (purple, control vector) into HEK293T cells. Cells were collected at 24, 48 and 72 h post-transfection using Lux Lysis Reagent (LLR) and luciferase activities were independently measured. Non-transfected cells without any vector were monitored for their signals as background. Lux activities were monitored by adding a 100 µL of reagent cocktail consisting of 5 µM FMN, 100 µM HPA, 10 µM decanal and 100 µM NADH in 50 mM sodium phosphate pH 7.0 into a cell lysate which was freshly mixed with 50 mU of C_1_ reductase. The luminescence signals were monitored for 10 sec with a 2 sec delay using an AB-2250 single tube luminometer. The Rluc activity was measured using *Renilla* Luciferase Assay Reagent according to manufacturer’s instructions. Data are presented as mean±SD of four biological replicates.

**
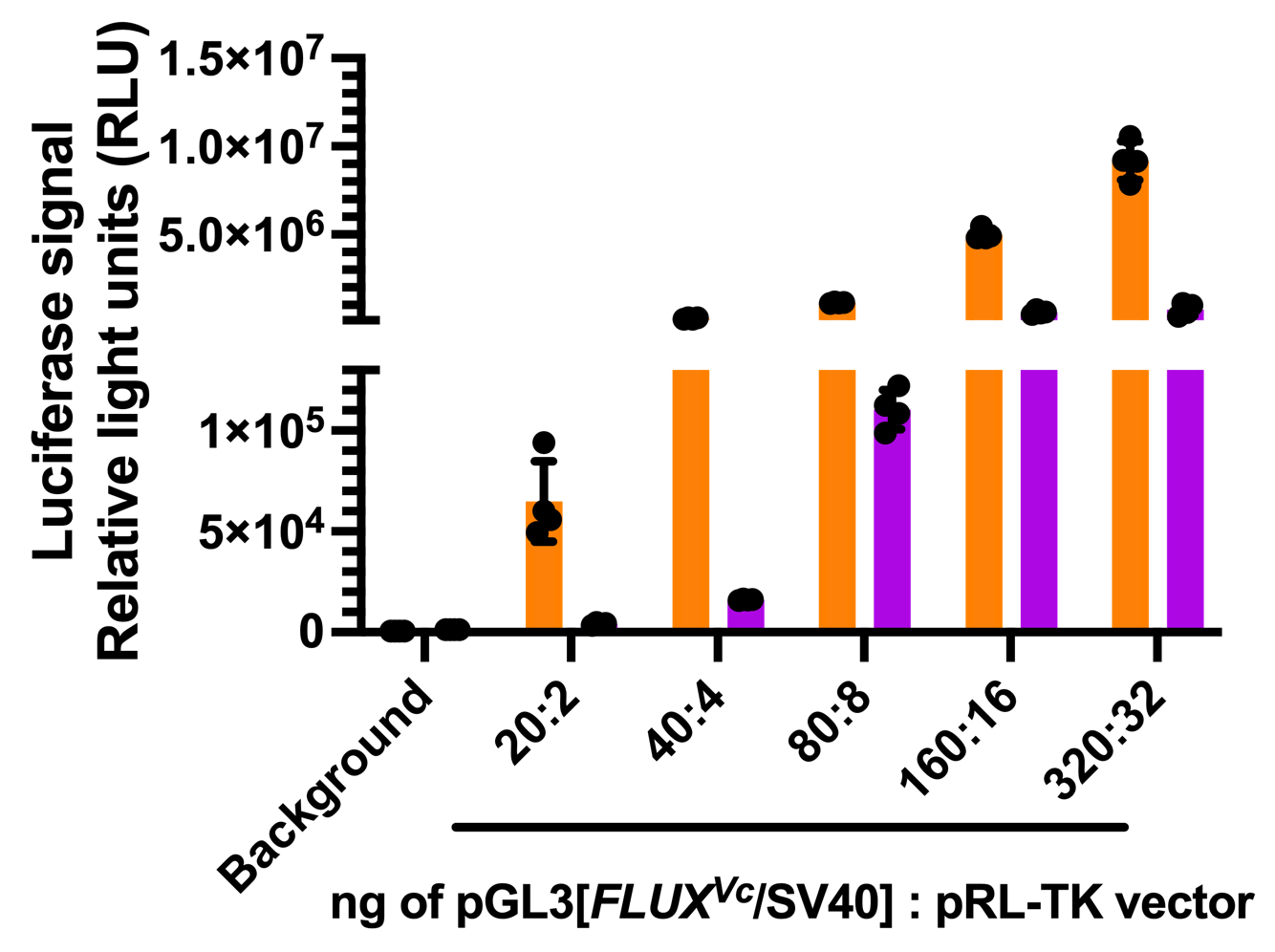
**

**Figure S9.** The FLUX^Vc^ signal (orange bar) from various amounts of pGL3 [*FLUX^Vc^*/SV40] vector and their Rluc internal control signal (Purple bar). Two-fold serial dilutions of pGL3 [*FLUX^Vc^*/SV40] vector from 320 to 20 ng were independent co-transfected with the pRL-TK vector as an internal control with a target:control vector ratio of 10:1 into HEK293T cells. The activity of FLUX^Vc^ was measured by adding a 100 µL of a cocktail reagent consisting of 5 µM FMN, 100 µM HPA, 10 µM decanal and 100 µM NADH in 50 mM sodium phosphate pH 7.0 into cell lysate freshly mixed with 50 mU of C_1_ reductase. The Rluc activity was measured using *Renilla* Luciferase Assay Reagent according to the manufacturer’s instructions. Luminescence signals were monitored for 10 sec with a 2 sec delay using an AB-2250 single tube luminometer. Data are presented as mean±SD of four biological replicates.

**
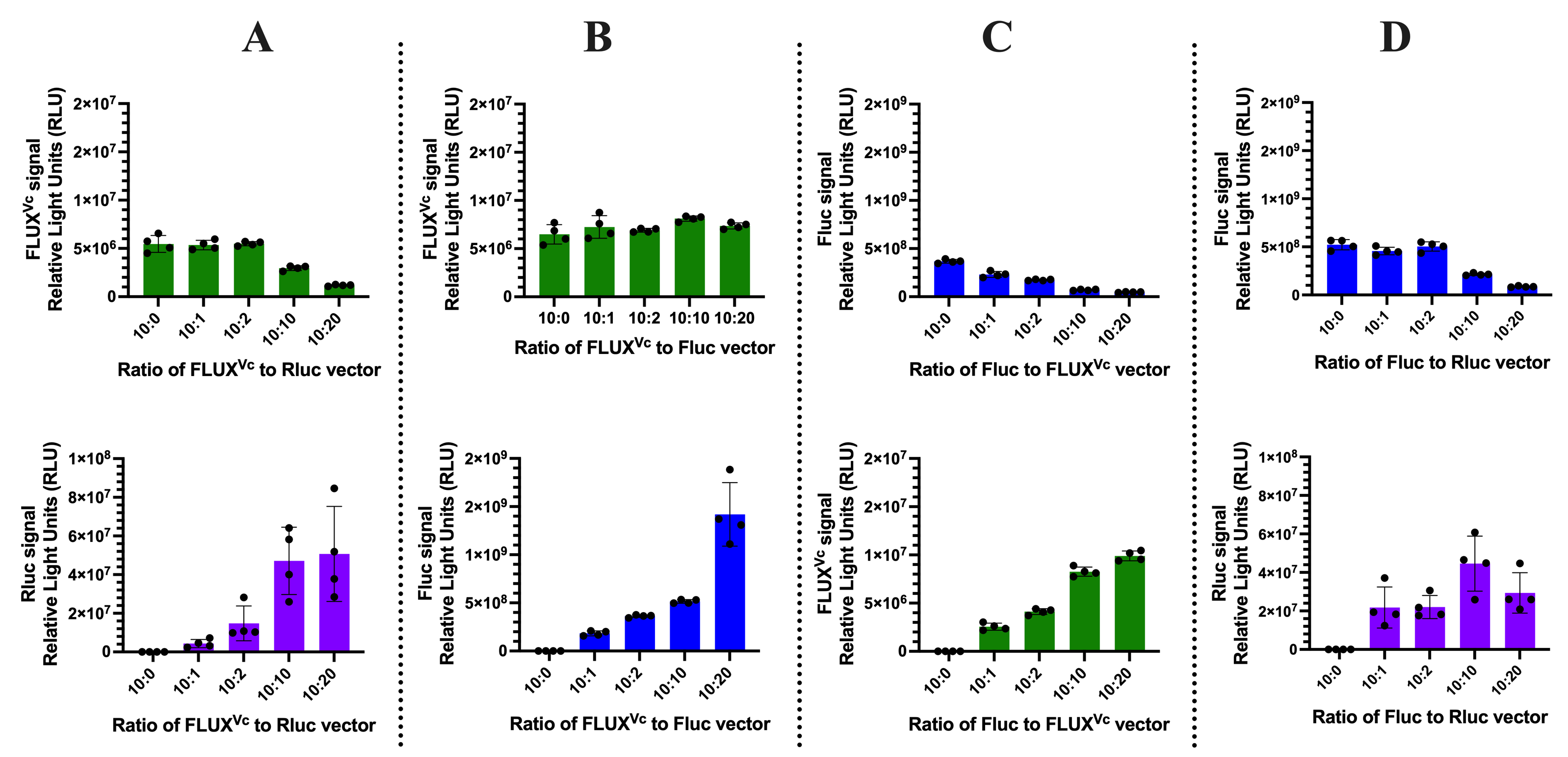
**

**Figure S10.** Individual signals of luciferase-based measurement at various ratios of targer:control vectors. Three types of luciferase vectors including pGL3[*luc*+/SV40] vector (blue), pGL3 [*FLUX^Vc^*/SV40] vector (green) and pRL-TK vector (purple) were used as either target or control vectors for four combinations including **A.** *FLUX^Vc^*/Rluc, **B.** *FLUX^Vc^*/Fluc, **C.** Fluc/ *FLUX^Vc^*, and **D.** Fluc/RLuc. The target vector was co-transfected with the control vector at various ratios ranging from 1:0, 1:0.1, 1:0.2, 1:1, and 1:2 into HEK293T cells. Cells were collected at 48 h post-transfection in either Passive Lysis Buffer (PLB) or Lux Lysis Reagent (LLR) and luciferase activities were independently measured. The activity of Lux was monitored by adding 100 µL of a cocktail reagent consisting of 5 µM FMN, 100 µM HPA, 10 µM decanal and 100 µM NADH in 50 mM sodium phosphate pH 7.0 into a cell lysate freshly mixed with 50 mU of C_1_ reductase. The luminescence signal was monitored for 10 sec with a 2 sec delay using an AB-2250 single tube luminometer. The Fluc activity was measured using firefly luciferase Assay Reagent according to the manufacturer’s instructions. Data are presented as mean±SD of four biological replicates.

**
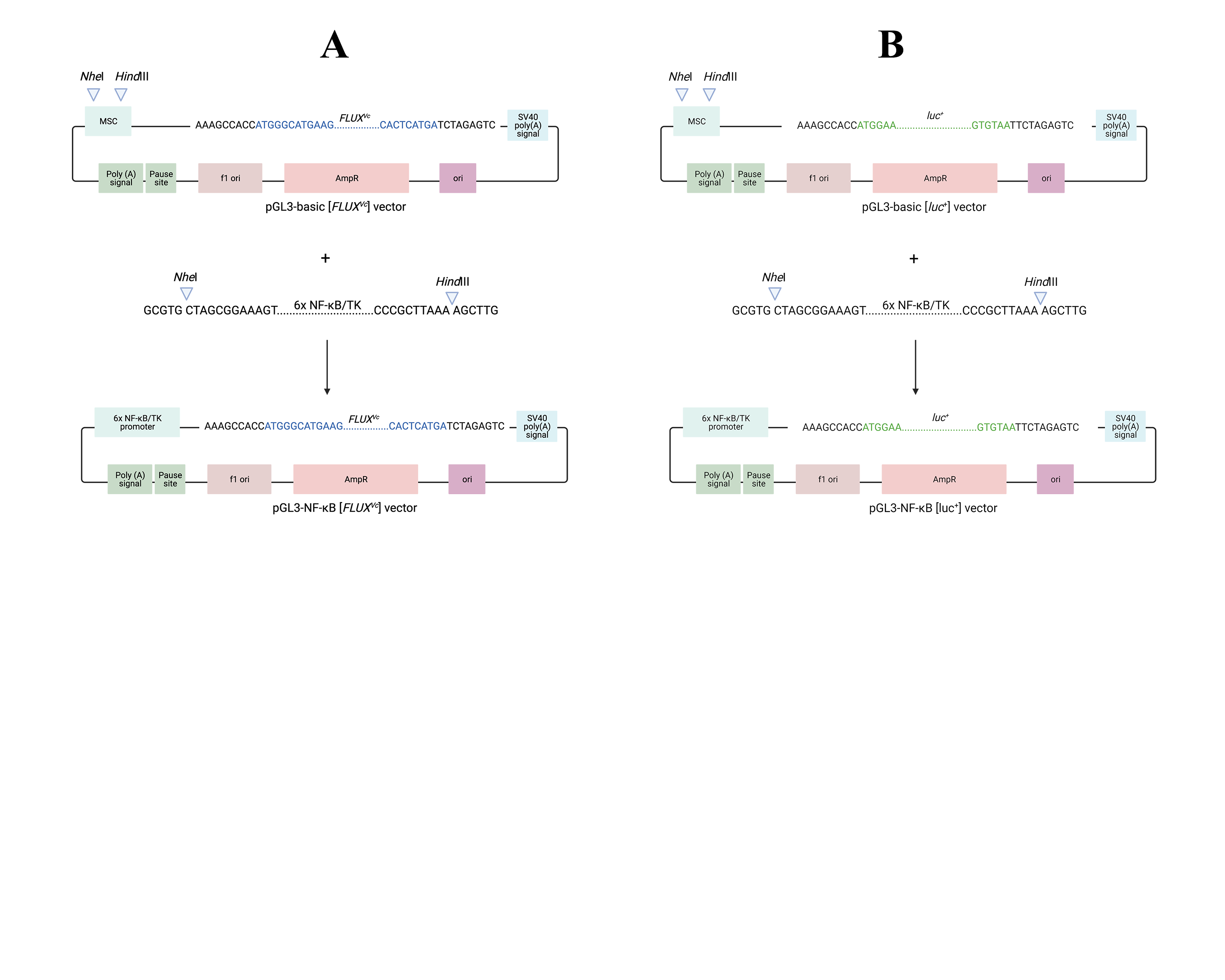
**

**Figure S11.** Maps of *FLUX^Vc^* and *luc+* gene reporters under the control of six tandem repeats of the NF-κB transcriptional element with the TK promotor. **A.** Construction processes of the pGL3-NF-κB [*FLUX^Vc^*/TK] vector and **B.** the pGL3- NF-κB [*luc+*/TK] vector. Construction processes of all vectors are similar. First, the pGL3 vector consisting of *FLUX^Vc^* or *luc*+ reporter gene was digested with *Nhe*I and *Hind*III restriction enzymes. Six tandem repeats of the NF-κB transcriptional element with TK promotor were amplified and digested by *Nhe*I and *Hind*III restriction enzymes. Then, the digested pGL3 vector was ligated with the digested *FLUX^Vc^* gene to obtain the pGL3-NF-κB reporter gene/TK vector consisting of either *FLUX^Vc^* or *luc*+ as a reporter gene.


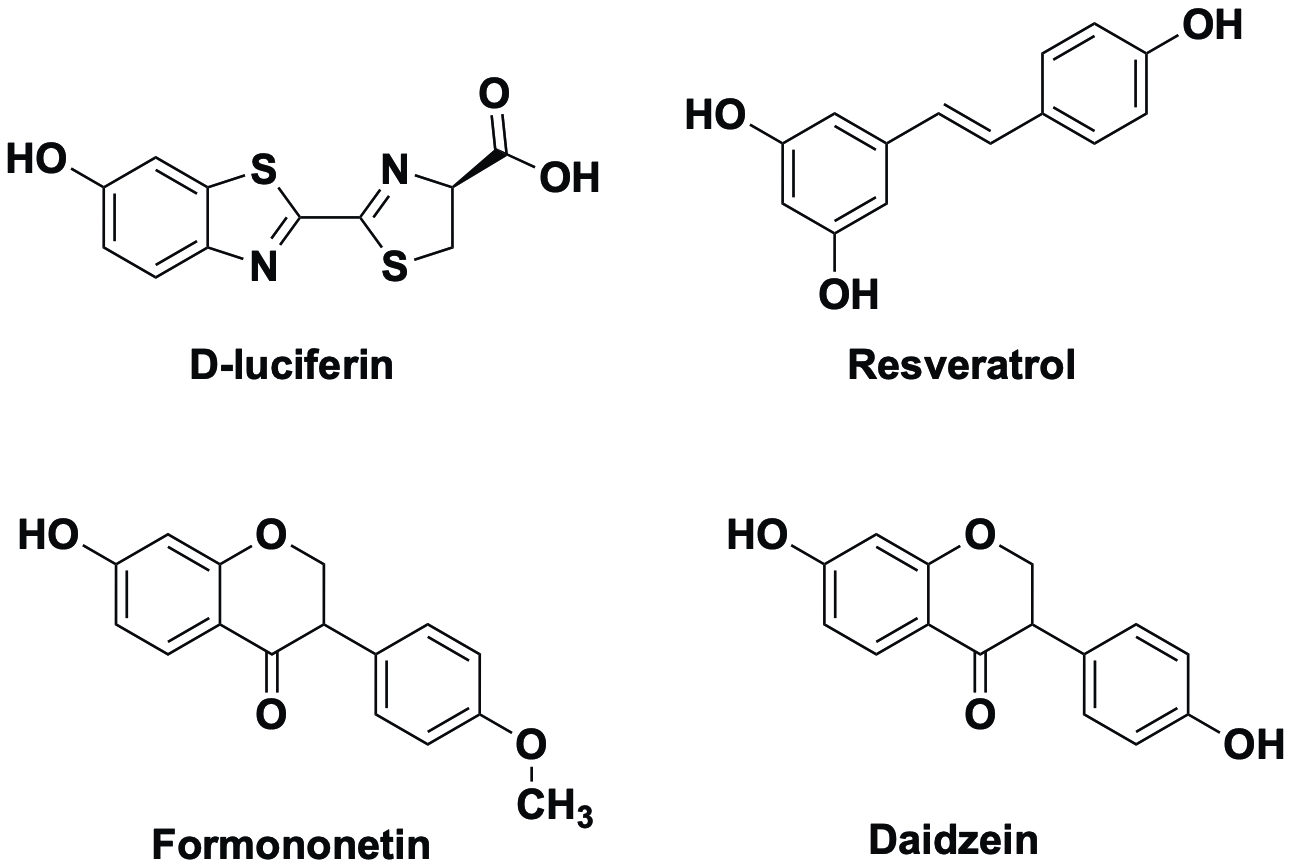


**Figure S12** Structures of D-luciferin and polyphenols including resveratrol, formononetin, and daidzein.
